# Structural and mechanistic insights into the transport of aristolochic acids and their active metabolites by human serum albumin

**DOI:** 10.1016/j.jbc.2024.107358

**Published:** 2024-05-22

**Authors:** Sergei Pomyalov, Conceição A. Minetti, David P. Remeta, Radha Bonala, Francis Johnson, Irina Zaitseva, Charles Iden, Urszula Golebiewska, Kenneth J. Breslauer, Gil Shoham, Viktoriya S. Sidorenko, Arthur P. Grollman

**Affiliations:** 1 Institute of Chemistry, The Hebrew University of Jerusalem, Jerusalem, Israel; 2 Department of Chemistry and Chemical Biology, Rutgers - The State University of New Jersey, Piscataway, New Jersey, USA; 3 Department of Pharmacological Sciences, Stony Brook University, Stony Brook, New York, USA; 4 Department of Chemistry, Stony Brook University, Stony Brook, New York, USA; 5 Department of Physiology, Stony Brook University, Stony Brook, New York, USA; 6 Department of Biological Sciences, Queensborough Community College, Bayside, New York, USA; 7 Rutgers Cancer Institute of New Jersey, New Brunswick, New Jersey, USA

**Keywords:** aristolochic acids, bioactivation, DNA adducts, environmental carcinogens, HSA-ligand interactions, fluorescence spectroscopy, isothermal titration calorimetry, X-ray crystallography

## Abstract

Aristolochic acids I and II (AA-I/II) are carcinogenic principles of *Aristolochia* plants, which have been employed in traditional medicinal practices and discovered as food contaminants. While the deleterious effects of AAs are broadly acknowledged, there is a dearth of information to define the mechanisms underlying their carcinogenicity. Following bioactivation in the liver, *N*-hydroxyaristolactam and *N*-sulfonyloxyaristolactam metabolites are transported *via* circulation and elicit carcinogenic effects by reacting with cellular DNA. In this study, we apply DNA adduct analysis, X-ray crystallography, isothermal titration calorimetry, and fluorescence quenching to investigate the role of human serum albumin (HSA) in modulating AA carcinogenicity. We find that HSA extends the half-life and reactivity of *N*-sulfonyloxyaristolactam-I with DNA, thereby protecting activated AAs from heterolysis. Applying novel pooled plasma HSA crystallization methods, we report high-resolution structures of myristic acid-enriched HSA (HSA_MYR_) and its AA complexes (HSA_MYR_/AA-I and HSA_MYR_/AA-II) at 1.9 Å resolution. While AA-I is located within HSA subdomain IB, AA-II occupies subdomains IIA and IB. ITC binding profiles reveal two distinct AA sites in both complexes with association constants of 1.5 and 0.5 · 10^6^ M^−1^ for HSA/AA-I *versus* 8.4 and 9.0 · 10^5^ M^−1^ for HSA/AA-II. Fluorescence quenching of the HSA Trp^214^ suggests variable impacts of fatty acids on ligand binding affinities. Collectively, our structural and thermodynamic characterizations yield significant insights into AA binding, transport, toxicity, and potential allostery, critical determinants for elucidating the mechanistic roles of HSA in modulating AA carcinogenicity.

Herbal remedies have been used for centuries to treat a variety of human diseases and chronic conditions. While traditional medicinal practices provide a reservoir of natural molecules for the development of conventional drugs (1, 2), credible data on the efficacy of crude herbal remedies is relatively sparse as the latter often elicit toxic side effects. The widespread use of aristolochic acids (AAs) in herbal recipes serves as a prime example that illustrates the overall magnitude of this problem (3, 4). AAs are natural products of *Aristolochia* plants with a long history of use in traditional Chinese and folk medicinal practices worldwide. These plants generate a variety of nitrophenanthrene carboxylic acids and aristolactams (5, 6), some of which are well-established kidney toxins and carcinogens (7). While AA-I and AA-II are the key carcinogenic species of *Aristolochia*, AA-I retains a dual role as a strong nephrotoxin (8).

During the past 2 decades, exposure to AAs has been associated with an outbreak of urinary tract diseases including urothelial cancer in Belgium (9, 10, 11) and Taiwan (12, 13, 14), endemic nephropathy in the Balkan Peninsula (15), renal cell carcinoma in Taiwan (16) and Croatia (17, 18, 19), bladder cancer in Europe (20) and Asia (21, 22), as well as hepatocellular carcinoma (23, 24) and intrahepatic cholangiocarcinoma (25, 26) throughout Asia (27, 28). A wealth of studies has provided significant evidence that DNA-adduct formation involving endogenous and/or exogenous compounds (29) leads to impairment of DNA replication which often escapes repair, thereby resulting in mutagenesis and carcinogenesis. While the mechanisms of AA-I nephrotoxicity are not well understood, the carcinogenicity of AA-I and AA-II is associated with the formation of aristolactam(AL)-DNA adducts (12, 15, 30), which are highly mutagenic and induce A to T mutations preferentially in the non-transcribed DNA strand within the 5′-CAG-3′ motif (31, 32, 33, 34, 35).

In order to bind DNA covalently, AAs require enzymatic activation as illustrated in Figure 1 and the pathways for biotransformation have recently been reviewed (36). Nitroreduction alone (37, 38) or followed subsequently by sulfonation generate *N*-hydroxyaristolactams (AL-NOH) and *N*-sulfonyloxiaristolactams (AL-NOSO_3_), respectively (39, 40, 41). AL-NOH and AL-NOSO_3_ undergo spontaneous heterolytic cleavage to form electrophilic cyclic nitrenium/carbenium ions, which are direct precursors of AL-DNA and AL-protein adducts. The enhanced reactivity of AL-NOSO_3_ relative to AL-NOH generates several hundred-fold more AL-DNA adducts than their respective precursors. A select number of enzymes are critical for mediating nitroreduction and sulfonation of AAs (36, 42, 43). An important yet underexplored route of bioactivation involves *N*-acetylation of AL-NOH by *N*-acetyltransferases yielding N-acetyloxyaristolactams (AL-NOAc) (41). Hereafter, we distinguish between aristolochic acids (AAs), *N*-hydroxyaristolactams (AL-NOH), and sulfonyloxyaristolactams (AL-NOSO_3_) by employing the designations AA-I, AA-II, AL-I-NOH, AL-II-NOH, AL-I-NOSO_3,_ and AL-II-NOSO_3_, respectively.

**Figure 1:**
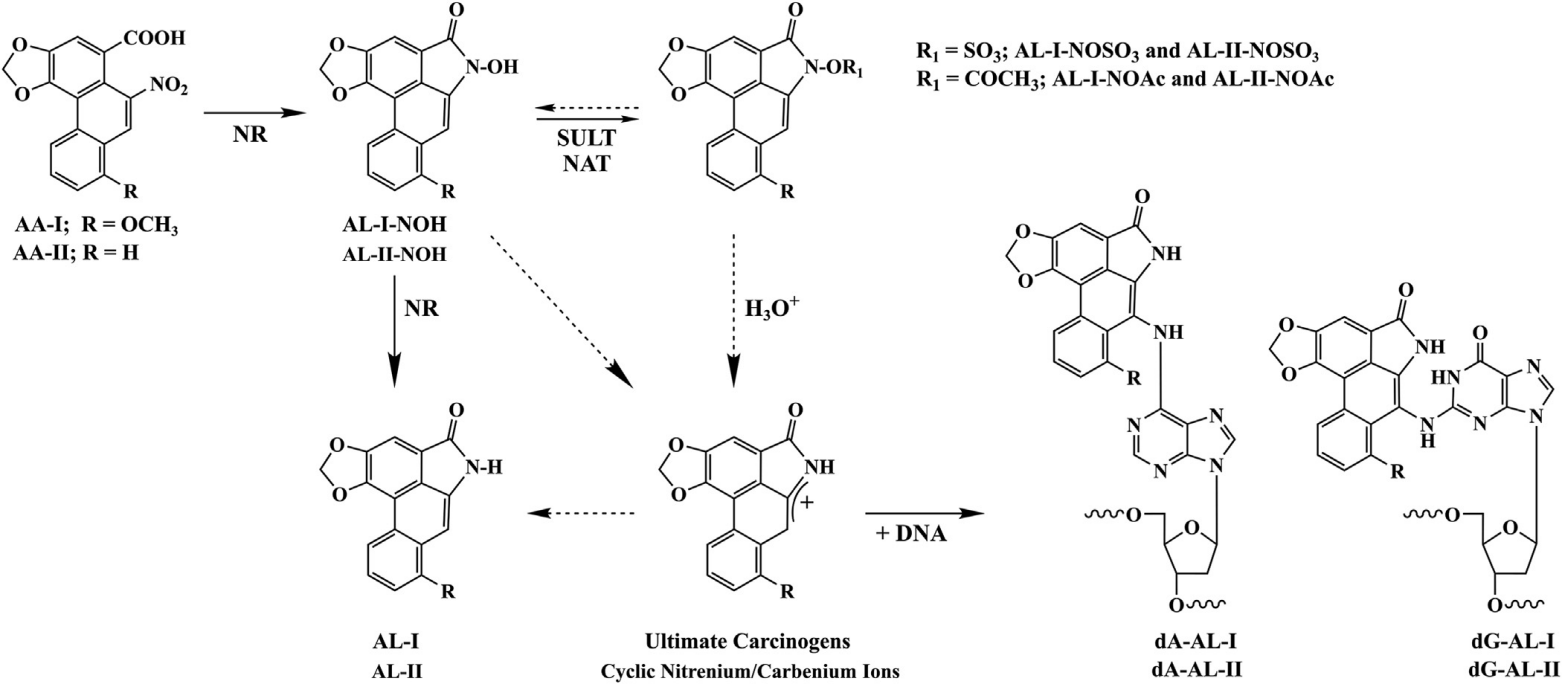
**Aristolochic acids and their bioactivation pathways.** Several enzymes including NQO1, CYPOR, XDH, and CYP1A/2 are responsible for the nitroreduction (NR) of AA-I and AA-II which generates the N-hydroxyaristolactams AL-I-NOH and AL-II-NOH. These metabolites may undergo further nitroreduction to form biologically inactive aristolactams AL-I and AL-II. Bioactivation *via* sulfonation catalyzed by SULT (sulfotransferases 1A1, 1A2, 1A3 and 1B1) or NO-acetylation by NAT (N-acetyltransferases 1 and 2) yields AL-NOSO_3_ or AL-NOAc species, respectively. The resultant N-sulfonyloxyaristolactams (AL-I-NOSO_3_ and AL-II-NOSO_3_) and N-acetyloxyaristolactams (AL-I-NOAc and AL-II-NOAc) readily undergo heterolytic cleavage forming cyclic nitrenium/carbenium ions. While these activated species are identical to those derived *via* decomposition of AL-I-NOH and AL-II-NOH, the latter are more stable than their respective esters and lead to DNA adduction at much slower rates. The genotoxicity of AAs is associated with formation of dA-(AL) and dG-(AL) aristolactam adducts. *Dashed arrows* signify non-enzymatic processes.

Using an integrated human liver-kidney organs-on-chips approach, we recently demonstrated that human hepatic cells produce active metabolites of AA-I, which can be distributed to the kidneys and presumably an array of organs (44). Although renal (45, 46) and multiple cell types (47, 48) are capable of generating active AA species, it appears that human liver cells contribute significantly to the overall carcinogenic effects observed in diverse tissues. Apart from its critical role in clearance and metabolic pathways, plasma protein binding may impact the distribution and effective toxicity of environmental compounds (49). Human serum albumin (HSA) is the most abundant blood protein and is well known to transport various endogenous and exogenous molecules (50) given its multisite binding properties and ability to accommodate a myriad of substances.

Considering the fundamental role of HSA in sequestering and transporting molecular species, there are a limited number of reports aimed at characterizing the binding of AAs to serum albumins (51, 52). HSA poses a unique advantage in that a single tryptophan residue (Trp^214^) is located in the IIA subdomain and serves as an intrinsic fluorophore. Analysis of ligand-induced fluorescence quenching profiles suggests the formation of a complex in which one AA-I molecule binds to HSA (52), presumably in subdomain IIA. While such reports provide evidence for interactions between AA-I and HSA, inspection of the experimental design protocol reveals that optimization of specific conditions coupled with more appropriate binding models is warranted. The current study addresses this deficiency by pursuing an experimental strategy that integrates calorimetric and spectroscopic techniques to facilitate acquisition of the requisite AA:HSA binding parameters.

Another limitation in binding studies is the frequent use of bulk commercial HSA preparations from pooled human plasma. These preparations often contain a significant ratio of cross-linked species as there is a single unpaired free thiol group (CySH34) in native HSA that is susceptible to various modifications including dimerization (50). While natural variations of CySH34 occur in the bloodstream due to the formation of mixed disulfide bonds with circulating amino acids such as cysteine and glutathione, dimeric CyS34-CyS34 albumin species are artificially generated during the purification of HSA from pooled human plasma. Furthermore, CySH34 is prone to form covalent bonds with electrophilic species of various xenobiotics (53). Such modified albumin species form crystals that diffract poorly (54) and there is insufficient evidence concerning how these modifications might impact ligand binding and/or the fluorescence quenching properties of HSA Trp^214^. Circumventing these limitations, we have generated a mercaptoalbumin-reduced form of HSA by incubating commercial protein samples under mild β-mercaptoethanol (BME) conditions employing a protocol applied successfully to study adduction between carcinogens and albumins *in vitro* (55, 56).

The majority of published studies focus on AA-I interactions with HSA which inevitably poses the query of whether AA-II exhibits comparable binding properties. In fact, prior to the current study, no high-resolution crystal structures have been reported for HSA bound to AAs, thereby restricting microscopic interpretations of molecular interactions based solely on spectroscopic binding data. As an illustrative example, competitive circular dichroism approaches employing biliverdin reveal that an AA-I/AA-II mixture binds to HSA subdomain IB (57) whereas binding-induced fluorescence quenching of Trp^214^ suggests that AA-I interacts with subdomain IIA (51, 52). Moreover, the characterization of interactions between HSA and active labile AA-metabolites remains sorely lacking and represents yet another fundamental area of interest requiring further exploration.

In this investigation, we apply mass spectrometric, fluorometric, and ^32^P-postlabelling approaches to reveal specific mechanistic aspects of the interactions between AAs and their active metabolites to bovine and human mercaptoalbumins. Crystallization of human serum mercaptoalbumin enriched with sodium myristate (herein designated as HSA_MYR_) in the absence and presence of AA-I or AA-II affords a detailed structural understanding of these molecular interactions. Collectively, our novel protocols allowed us to obtain several high-resolution crystal structures including HSA_MYR_ (PDB ID 8RCP, 1.9 Å), HSA_MYR_ bound to AA-I (HSA_MYR_/AA-I, PDB IDs 8RGK, 8RGL, 1.9 Å), and HSA_MYR_ bound to AA-II (HSA_MYR_/AA-II, PDB ID 8RCO, 1.9 Å). Employing a complementary array of calorimetric and spectroscopic techniques, we characterized the requisite binding properties for both AA-HSA complexes. Our integrated biophysical approach unequivocally demonstrates that AA-I and AA-II interactions with HSA are best described by a two-site binding model in which the resultant complexes are comprised of two AA molecules bound to an HSA monomer. We interpret the AA-I and AA-II binding parameters within the context of specific interactions gleaned *via* structural elucidation of intermolecular contacts in the AA-HSA complexes.

## Results

### Serum albumins protect *N*-sulfonyloxyaristolactam from decomposition and extend its reactivity with DNA

**Figure 2:**
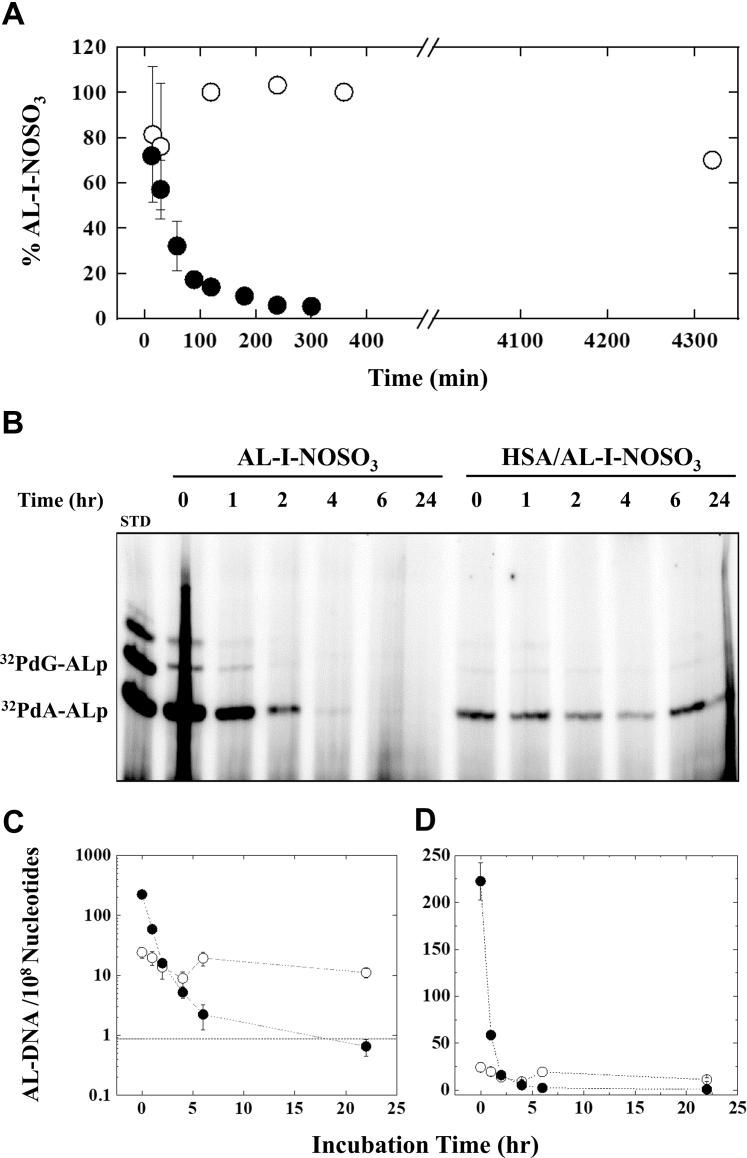
**Impactof serum albumins on the stability and activity of AL-I-NOSO**_**3**_**.***A*, ESI-LC/MS stability of AL-I-NOSO_3_ in the absence (*closed circles*) and presence (*open circles*) of BSA. AL-I-NOSO_3_ incubated for various time intervals followed by protein precipitation and evaluation of compound stability by mass spectrometry as described in Experimental procedures. *B*, representative PAGE of the reaction mixtures involving ^32^P-postlabelling technique to visualize AL-DNA (refer to Experimental procedures) in the absence (*left*) and presence (*right*) of HSA for various time intervals prior to addition of DNA as indicated. The first lane (STD) represents a mixture (30 fmol each) of standard (dA-AL-II and dG-AL-II) oligonucleotides. The bands above ^32^PdG-AL-phosphate correspond to digestion and labelling artifacts that are occasionally observed for certain batches of kinases and phosphodiesterases. *C* and *D*, gel analysis and quantification in the absence (*closed circles*) and presence (*open circles*) of HSA depicted in logarithmic and linear scales, respectively. AL-DNA levels represent the sum of all bands detected in the gel. Each datapoint represents the mean and standard deviation obtained from 3 to 6 DNA reactions. *Dashed line* indicates the minimal detection level.

Our initial studies assessed whether the presence of serum albumin affects the half-life of AL-I-NOSO_3_ in solution by monitoring the impact of BSA on its stability as a function of time. Towards this end, we incubated AL-I-NOSO_3_ in the absence and presence of 600 μM BSA over time periods spanning minutes to several days. At the prescribed time intervals, we combined sample aliquots with ice-cold methanol to precipitate BSA. Analysis *via* electrospray ionization liquid chromatography mass spectrometry (ESI-LC/MS) facilitated comparison of free AL-I-NOSO_3_ in the supernatant relative to its amount at zero time as illustrated in Figure 2*A*. In the absence of albumin, half of AL-I-NOSO_3_ decomposes within 45 min to non-active forms including aristolactam I, AL-I-NOH and other unidentified species. In contrast, AL-I-NOSO_3_ remains nearly intact when incubated in the presence of BSA for 24 h. A similar approach undertaken for AL-I-NOAc reveals that the acetylated form apparently binds to the walls of test tubes and equipment within minutes, thereby impeding estimation of its half-life (data not shown).

We subsequently extended our studies to assess the impact of HSA on metabolite activity. In principle, we surmised that AL-I-NOSO_3_ forms fewer AL-DNA adducts in the presence of HSA as a direct consequence of drug binding. Concurrently, the activity of AL-I-NOSO_3_ is not expected to degrade significantly when incubated with HSA over various time periods prior to DNA addition. Finally, we anticipated that AL-I-NOSO_3_ incubated *sans* protein might experience a rapid decline in DNA reactivity over time. The experimental results presented in Figure 2, *B*–*D* support our conjecture. Upon diluting AL-I-NOSO_3_ in phosphate buffer (zero time point), this AA metabolite forms 225 AL-DNA adducts per 10^8^ nucleosides following incubation with DNA for 30 min. Under similar conditions, a solution of AL-I-NOSO_3_ pre-incubated in the presence of HSA exhibits ten times less reactivity upon exposure to DNA resulting in the formation of 25 AL-DNA adducts per 10^8^ nucleosides. When AL-I-NOSO_3_ is allowed to incubate in buffer alone for various time intervals prior to addition of DNA, its activity declines tenfold within 2 h, decreasing to the level of detection between 6 and 24 h. Strikingly, the activity of an AL-I-NOSO_3_/HSA mixture remains relatively constant fluctuating near its zero time point level for 24 h. Adduct formation in the presence of HSA corresponds to ∼10% of the control (*i.e.*, AL-I-NOSO_3_ *sans* albumin), implying that ∼90% of the metabolite remains “sequestered” under equilibrium conditions. Collectively, these results demonstrate that AL-I-NOSO_3_ binding to serum albumin protects the AA-I sulfonated metabolite from decomposition and thereby precludes DNA adduct formation *in vitro*.

### HSA fluorescence quenching assays

Ligand-induced quenching of the intrinsic HSA fluorescence has been assessed by monitoring a single Trp^214^ residue located within the IIA binding cavity to derive equilibrium dissociation constants (*K*_D_) for numerous endogenous and exogenous molecules including aristolochic acids (51, 52). These studies generally focus on commercial preparations of HSA that do not specify the percentage of fatty acids, amount of CyS34-CyS34 protein dimers, and the ratio of modified CySH34 in the samples (58). Addressing this deficiency, we prepared mercaptoalbumins from HSA_A3782_ and HSA_A8763_, consisting of fatty acid-free and limited fatty acid-containing HSA, respectively. We subsequently employed these well-defined HSA samples to conduct comparative fluorescence quenching studies in the presence of AA-I, AA-II, and their respective metabolites while concurrently evaluating the impact of fatty acid content on relative binding affinities. The fluorescence quenching profiles are presented in Figs. S1 and S2, and the resultant data are summarized in Table S1.

The differential impact of fatty acids on HSA interactions with AAs and their active metabolites has been assessed by comparing the quenching profiles of HSA_A3782_ and HSA_A8763_ in the presence of AA-I, AA-II, AL-I-NOH, AL-II-NOH, AL-I-NOSO_3_, and AL-I-NOAc as observed in Fig. S1. Relative to unmodified AA-I, the corresponding metabolites with exception of AL-I-NOH exhibit an increased apparent dissociation constant (K_Dapp_) that is effectively abrogated in the presence of fatty acids as summarized in Table S1. Interestingly, the K_Dapp_ observed for AA-II binding to defatted protein is higher than that of AA-I yet significantly decreased in the presence of fatty acids. Conversely, the AA-II-NOH metabolite exhibits a higher binding affinity to HSA in the absence of fatty acids. The presence of fatty acids therefore differentially impacts ligand-dependent HSA quenching by either decreasing (AL-I-NOSO_3_, AA-II) or increasing (AL-II-NOH) the apparent dissociation constant as depicted in Fig. S2. These findings might reflect binding to distinct sites within HSA and/or varying degrees of interdependence between ligand and fatty acid interactions. Since HSA quenching profiles of Trp^214^ yield concentration-dependent K_Ds_ (data not shown), the latter precludes accurate determination of binding parameters under these experimental conditions (59). Consequently, the characterization of AA-HSA affinity constants and binding stoichiometry requires the use of alternative approaches that do not rely on intrinsic fluorescent probes. The following section describes application of isothermal titration calorimetry (ITC) to monitor AA-HSA interactions and determine the number of binding sites and respective affinities.

### Characterization of AA-HSA interactions *via* isothermal titration calorimetry

We have assessed the binding affinities of AA-I and AA-II for HSA_A3782_ by conducting a series of ITC experiments. Acquisition of binding isotherms at varying AA (100–200 μM) and HSA (5–10 μM) concentrations facilitated optimization of solution conditions by minimizing incompetent species and ensuring that ligand aggregation and/or precipitation does not appreciably impact the calorimetric profiles. An initial analysis of the AA-HSA binding isotherms using conventional fitting programs provides a reasonable estimate for the binding stoichiometry of two AA ligands per HSA molecule. Moreover, inspection of the ITC profiles reveals that formation of the AA-HSA complex is exothermic and characterized by micromolar binding affinities. Given the challenges associated with baseline assignments, we subsequently employed the NITPIC/SEDPHAT/GUSSI software suite (60) to ensure unambiguous analysis of the binding isotherms. Singular value decomposition yields baseline-corrected association data that are analyzed *via* a two-site binding model to derive macroscopic affinities for AA-HSA interactions in accordance with the following relation:

A+B+B↔[AB]+B↔ABB

where A represents the HSA macromolecule and B represents ligand AA-I or AA-II. This binding model implies that both sites are occupied with all possible ligation states coexisting in equilibrium.

**Figure 3:**
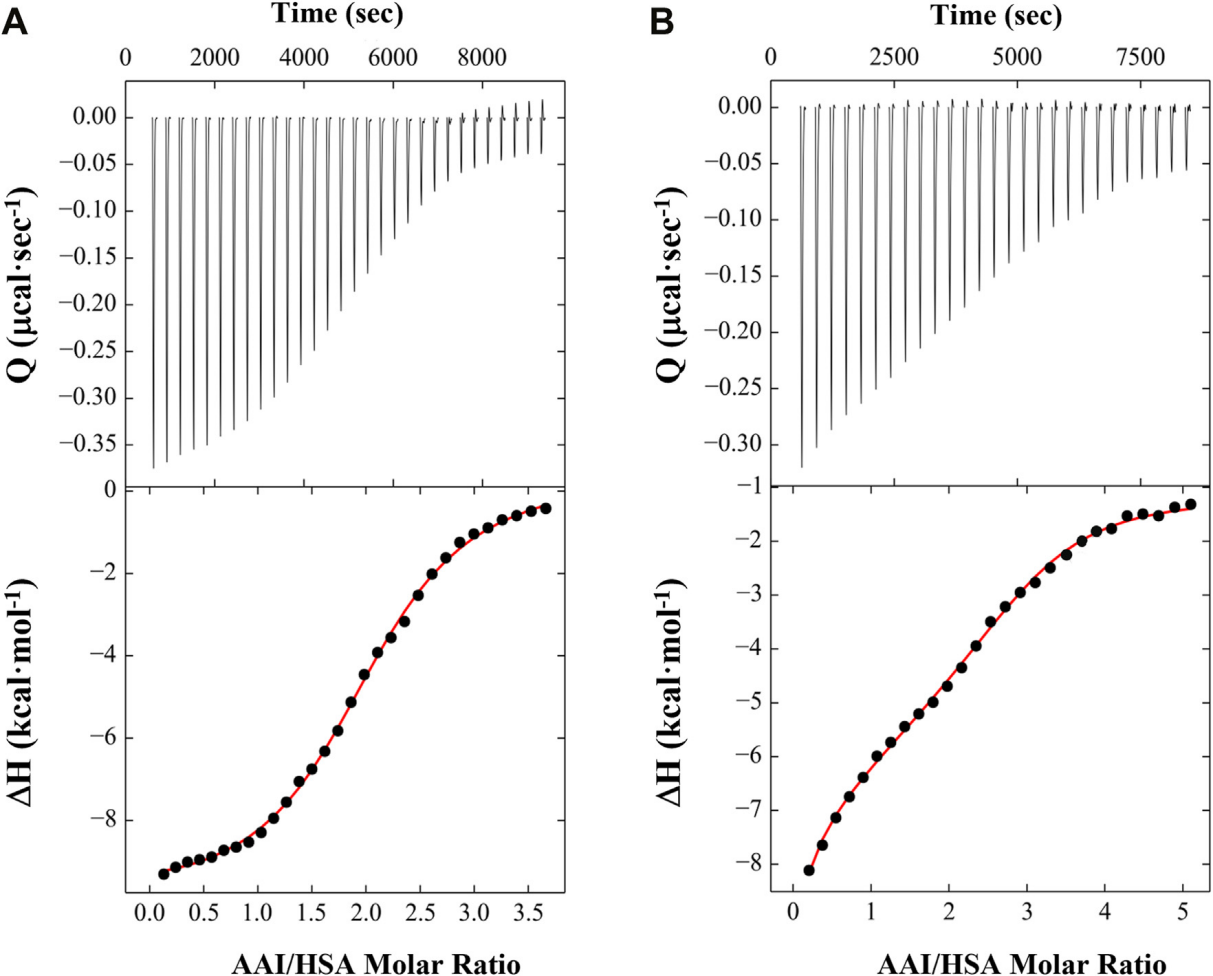
**Characterization of AA-HSA binding energetics *via* isothermal titration calorimetry.** Representative ITC profiles for the association of AA-I (*A*) and AA-II (*B*) with HSA. The *upper panels* correspond to exothermic reaction heats generated upon titration of AAs into HSA. The *lower panels* depict integrated binding isotherms (*black circles*) expressed as a function of AA:HSA ratios. The resultant data are fit to a model of two binding sites (*red lines*) to derive the macroscopic association constants as described in Experimental procedures (Refer to Table 1).

Applying this experimental strategy to characterize AA-I and AA-II interactions with HSA, we have determined macroscopic affinity constants for a family of binding isotherms acquired at 25 °C as illustrated in Figure 3. The formation of AA-HSA complexes is characterized by moderate binding affinities ranging from 10^5^ to 10^6^ M^−1^. Inspection of the calorimetric profiles and resultant parameters reveals that both AA ligands exhibit two-site binding modes with macroscopic affinity constants (K_a_) of 1.5 · 10^6^ M^−1^ and 5.4 · 10^5^ M^−1^ for AA-I *versus* 8.4 · 10^5^ M^−1^ and 9.0 · 10^5^ M^−1^ for AA-II as summarized in Table 1. The corresponding dissociation constants (K_D_) are 0.69 and 1.87 μM for AA-I compared with 1.19 and 1.11 μM for AA-II, respectively. Collectively, our global analysis employing a two-site binding model demonstrates that the AA-HSA interactions involve two distinct sites which might represent independent or cooperative events. This finding is consistent with the unique nature of HSA multisite binding properties, particularly when comparing molecular interactions for the AA ligands at subdomains IB and IIA as deduced *via* structural analysis.

**Table 1:** Binding parameters derived for the association of AA-I and AA-II with HSA

Ligand	Binding site	K_a_ · 10^6^ (M^−1^)	K_D_ (μM)	ΔG° (kcal·mol^−1^)
AA-I	Site 1	1.50 ± 0.18	0.69 ± 0.08	−8.41 ± 1.00
	Site 2	0.54 ± 0.04	1.87 ± 0.12	−7.82 ± 0.58
AA-II	Site 1	0.84 ± 0.07	1.19 ± 0.19	−8.10 ± 0.67
	Site 2	0.90 ± 0.10	1.11 ± 0.11	−8.12 ± 0.82

Binding affinities are derived from nonlinear fits of the ITC profiles (Fig. 3) to a model comprised of two sites which yields the requisite macroscopic association constants. Binding data correspond to average values and standard deviations determined for a minimum of three ITC measurements performed in 10 mM NaPO4 (pH 7.5) at 25 °C. The resultant binding free energies are calculated *via* the following standard thermodynamic relation: ΔG° = −RTlnK_a_ with T = 298 K.

Attempts to derive microscopic association constants *via* a non-symmetric two-site model suggest a degree of cooperativity associated with AA-HSA interactions. Further assessment employing orthogonal approaches and single-site displacement titrations are required to unambiguously characterize intrinsic microscopic association constants. Future studies are therefore warranted to evaluate the potential role of allosteric effects on crosstalk amongst multiple sites and between various ligands including endogenous and exogenous compounds such as metals (*e.g.*, Zn^2+^) (61) and drugs (*e.g.*, benzodiazepine) (62) in the presence of free fatty acids. This is particularly critical in view of reports implicating fatty acids as triggering HSA conformational switches (63). It is worth noting that the calorimetric and crystallographic data presented herein are acquired on defatted and myristic-acid containing HSA preparations, respectively. Consequently, structural-energetic correlations are limited as a complete thermodynamic characterization is required to evaluate the overall impact of fatty acids on AA-HSA binding energetics.

### Structural analysis of HSA_MYR_ and HSA_MYR_/AA complexes

As a prerequisite to elucidating specific binding interactions between HSA and AA species, we sought to crystallize and determine the detailed molecular structure of HSA in the absence and presence of AA-I and AA-II. Toward this end, we prepared monomeric HSA species enriched with sodium myristate to obtain high-quality crystals of HSA. This approach allowed us to determine the structure of HSA_MYR_ and its complexes with AA-I and AA-II at a resolution of 1.9 Å, arguably amongst the highest resolution HSA structures reported to date. The crystal structures obtained under these conditions are affected by the presence of strong translational non-crystallographic symmetry (tNCS). Remarkably, the presence of tNCS in these structures did not complicate molecular replacement due to the availability of HSA models yet may account at least in part for the elevated R-factor statistics. Refinement parameters of HSA_MYR_, HSA_MYR_/AA-I, and HSA_MYR_/AA-II with their respective accession numbers are presented in Table 2. The individual B-factors and corresponding ligand occupancies are summarized in Table S2.

**Table 2:** Representative data collection and refinement parameters for crystal structures

Protein	HSA_MYR_ complex	HSA_MYR_/AA-I complex	HSA_MYR_/AA-I complex (2 AA-I)	HSA_MYR_/AAII complex
Data Collection and Refinement				
Beamline	DIAMOND-i03	ESRF-BM30	ESRF-BM30	DESY-P13
Processing software	DIALS	XDS	XDSGUI	DIALS
Space group	*P12*_*1*_*1*	*P12*_*1*_*1*	*P12*_*1*_*1*	*P12*_*1*_*1*
Wavelength (Å)	0.9762	0.9798	0.9798	0.9786
Rotation range (°)	293	227	227	259
Oscillation range Δφ (°)	0.1	1	1	0.1
Unit cell dimensions (Å)				
a	95.728	94.397	95.247	94.821
b	38.562	37.872	38.189	38.671
c	183.557	180.248	181.918	180.841
Resolution (Å)	88.79–1.90 (1.93–1.9)^a^	45.56–1.9 (1.968–1.9)	45.98–1.93 (1.93–1.90)	174.74–1.9 (1.93–1.9)
No. of reflections				
Total	523,824 (19,220)	411,167 (36,809)	409,672 (15,687)	464,097 (22,161)
Unique	102,879 (4432)	97,365 (9562)	99,387 (4618)	101,166 (4813)
Multiplicity	5.1 (4.3)	4.2 (3.8)	4.1 (3.4)	4.6 (4.6)
Completeness (%)	98.7 (85.6)	98.8 (98.65)	98.5 (95.0)	99.3 (95.4)
<I/σ(I)>	7.5 (0.7)	10.93 (2.53)	10.9 (2.7)	6 (0.9)
Wilson B-factor	31.7	26.35	23.85	32.63
R_meas_ (%)	0.095 (1.341)	0.08848 (0.5427)	0.087 (0.542)	0.090 (1.371)
R_pim_ (%)	0.042 (0.616)	0.04216 (0.2654)	0.041 (0.275)	0.041 (0.618)
CC half	0.996 (0.599)	0.996 (0.796)	0.997 (0.802)	0.995 (0.633)
*R*_work_ (%)	0.2243 (0.3857)	0.2402 (0.3421)	0.2305 (0.3231)	0.2304 (0.3371)
(no. of reflections)	102,482 (9781)	97,330 (9557)	99,346 (9497)	99,375 (9864)
*R*_free_ (%)	0.2687 (0.4471)	0.2894 (0.3600)	0.2807 (0.3686)	0.2710 (0.3708)
(no. of reflections)	5037 (449)	4931 (492)	5016 (480)	4715 (467)
Refined Model				
No. of protein chains	2	2	2	2
No. of residues	1164	1164	1164	1164
No. of ligands atoms	724	685	610	680
No. of solvent molecules	659	529	544	594
No. of atoms (overall) (non-H)	10,100	9969	9974	10,055
<B> factor (Å^2^) overall	40.19	41.35	41.72	43.86
<B> factor (Å^2^)				
Macromolecules	39.78	41.53	41.96	43.55
Ligands	45.44	41.78	43.63	45.50
FA	51.07	49.54	50.52	51.62
AA	--	26.07	46.60	43.08
Solvent	43.83	38.11	36.86	47.77
Mean ligand occupancy				
FA	0.93	0.93	0.92	0.88
AA		0.88	0.92	0.89
Ramachandran plot				
Favored (%)	98.36	97.50	98.02	98.10
Allowed (%)	1.64	2.50	1.98	1.90
Disallowed (%)	0.00	00.20	0.00	0.00
RMS deviation				
Bond lengths (Å)	0.010	0.004	0.008	0.004
Bond angles (°)	1.10	0.66	0.90	0.57
PDB code	8RCP	8RGK	8RGL	8RCO

a Values for highest-resolution shell appear in parentheses.

**Figure 4:**
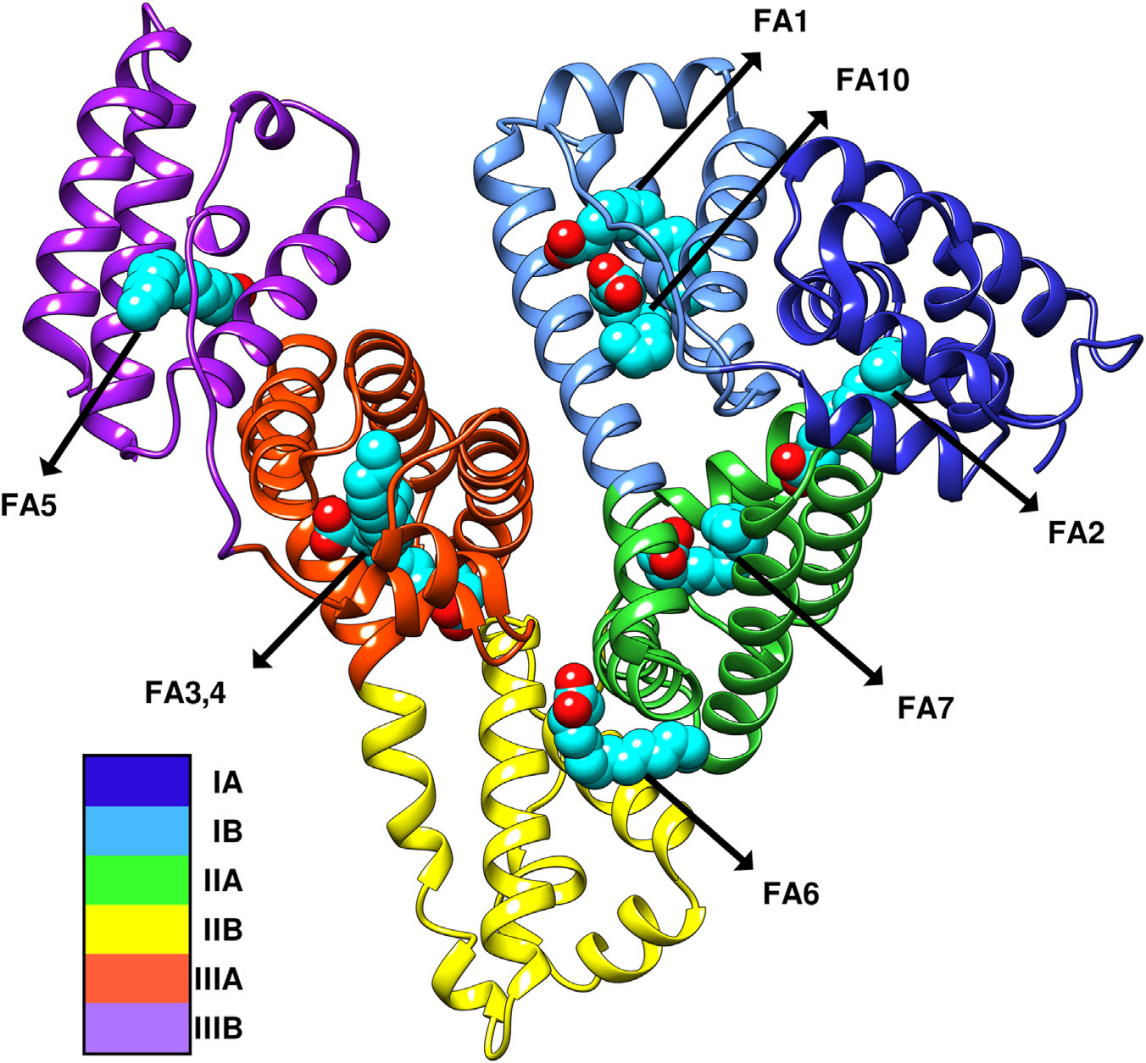
**Structure of HSA**_**MYR**_**complex.** A secondary structure ribbon representation of the HSA_MYR_ complex colored by its individual subdomains. Fatty acid (FA) and drug binding sites are designated by *arrows* with the myristate ligands colored *light blue*. Several of the FA and drug binding sites are specified according to common designations: FA7 = Drug Site I = Subdomain IIA; FA3,4 = Drug Site II; FA1,10 = Drug Site III = Subdomain IB.

The structure of HSA_MYR_ contains two protein molecules in the asymmetric unit, adopting a conformation that is comparable to HSA_MYR_ structures reported previously (64). Under these crystallization conditions, myristic acid ligands occupy the known HSA primary fatty acid binding sites (FA1-7) as illustrated in Figure 4. In contrast with the majority of HSA_MYR_ structures reported to date, we observe a second myristate molecule situated at the lip of subdomain IB in addition to the classical FA site 1 located between Y161 and Y138. This finding suggests the presence of two distinct FA sites within subdomain IB as depicted in Fig. S3. The binding of an additional myristate molecule is characterized by a refined occupancy of 0.82 to 0.83 (Table S2). This value is somewhat lower compared to the average occupancy of classical sites (*i.e.*, 0.94–0.95), albeit not significantly different than the average occupancy of 0.88 for neighboring FA1 in published structures. Previous studies report that under specific experimental conditions including the presence of short-chain and/or an excess of fatty acids (65), two additional secondary sites (*i.e.*, FA8 and FA9) are usually detected in the gap between subdomains IA-IB-IIA and IIB-IIIA-IIIB (66, 67, 68) yet are absent in our crystallographic analysis of HSA_MYR_. Nevertheless, in view of the existing designations (FA1–FA9) and our finding of a novel fatty acid binding site located in subdomain IB within the vicinity of H146, we tentatively assign the latter as FA10 (refer to Figs. 4 and S3).

### Structure of the HSA_MYR_/AA-I complex

**Figure 5:**
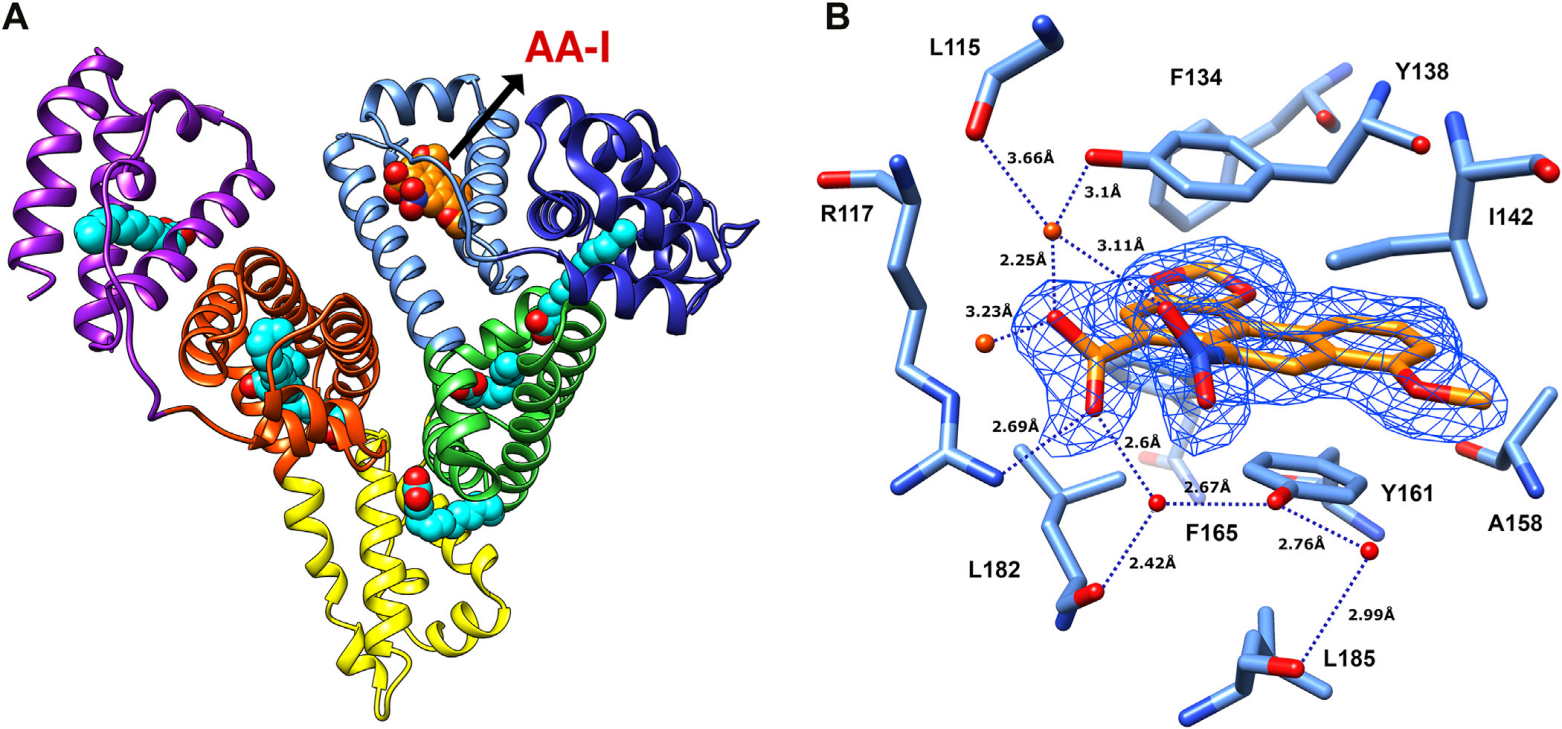
**Structure of HSA**_**MYR**_**/AA-I complex.***A*, AA-I occupies FA site 1 in subdomain IB. *B*, residues and interactions surrounding AA-I bound in the pocket of subdomain IB (Chain A). The *2Fo-Fc* electron density of AA-I is contoured at 1σ (*blue mesh*) with hydrogen bonding distances (*blue dotted lines*), adjacent water molecules, and residues.

The structure of HSA_MYR_/AA-I reveals that AA-I occupies the main pocket located in subdomain IB, thereby replacing both myristate ligands (*i.e.*, FA1, FA10) in HSA_MYR_ as illustrated in Figure 5*A*. It is important to note that the crystallographic asymmetric unit of this crystal form includes two independent HSA molecules (*i.e.*, HSA protein chains A and B as described above). While the electron density of AA-I bound to one of the HSA chains is evident in the crystal structure as depicted in Figure 5*B*, the second HSA chain contains what resembles AA-I and myristate bound at a mixed occupancy. Therefore, two structures of the HSA_MYR_/AA-I complex have been modeled as often performed in such cases. Specifically, one structure contains AA-I bound to subdomain IB of HSA chain B and a myristate molecule in the respective position of HSA chain A (PDB ID 8RGK), whereas a second structure includes AA-I modeled in the subdomain IB binding pocket of each chain (PDB ID 8RGL).

In both instances, AA-I molecules are bound to subdomain IB (FA1 site) in its open conformation, comparable to that observed for HSA bound to myristate or hemin (69, 70). In its bound state, AA-I is almost completely buried within the binding pocket, while only the carboxylate and nitro groups protrude slightly towards the solvent (Fig. S4*A*). The electron density of AA-I (Fig. S5, *A* and *B*) is sufficiently clear to demonstrate its exact orientation and position within the binding site. The methylenedioxy ring of AA-I faces the F134 HSA residue and is wedged deep inside this pocket. The methoxy group of AA-I points in the opposite direction and occupies an otherwise empty region of the IB binding cleft (Fig. S4*B*). This region houses a portion of the bulky hemin ring in previously characterized HSA-hemin complex structures (70, 71) (refer to Fig. S9*A*). HSA residue R117 located at the “lip” of this pocket forms a hydrogen bond with the carboxylate moiety of AA-I (Figs. 5*B* and S6*A*). The latter is hydrogen bonded with Y138 through a bridging water molecule located at a reasonable distance from the nitro group of AA-I in chain A.

The carboxylate group of AA-I appears to be engaged in an additional water bridge with Y161 and the main chain carbonyl of L182 (Figs. 5*B* and S6*A*). This tight hydrogen bonding arrangement resembles that of the carboxylate moiety in myristate (FA1) bound to subdomain IB of HSA_MYR_. Interestingly, R186 forms a hydrogen bond with the nitro group of AA-I in one chain, whereas this residue and K190 in the second chain face out towards the solution and do not appear to form specific interactions with the nitro group despite their relative proximity. These local differences between the two HSA chains might be linked at least partially to the mixed occupancy of AA-I in one chain as discussed above. Aside from interactions formed by the carboxylate and nitro groups with surrounding HSA residues and water molecules, AA-I is tightly bound by hydrophobic interactions with aliphatic and aromatic residues that line the inner side of this pocket. Two of the HSA tyrosines in this region (Y138 and Y161) are splayed and AA-I occupies the space between these residues (Fig. 5*B*). Specifically, AA-I is sandwiched between Y138 and Y161 in a parallel π-stacking configuration. F134 and F165 surround AA-I from the direction of the methylenedioxy ring, while A158, I142, and F157 flank AA-I on its methoxy group side (Fig. S6*A*). The combination of tight hydrogen bonding and hydrophobic interactions described above is consistent with the strong binding and fixed orientation of AA-I in the FA1 site. Collectively, these findings coupled with a high refined occupancy of 0.91 are presumably the reason that such binding is clearly observed in the HSA_MYR_/AA-I crystal structure.

### Structure of the HSA_MYR_/AA-II complex

**Figure 6:**
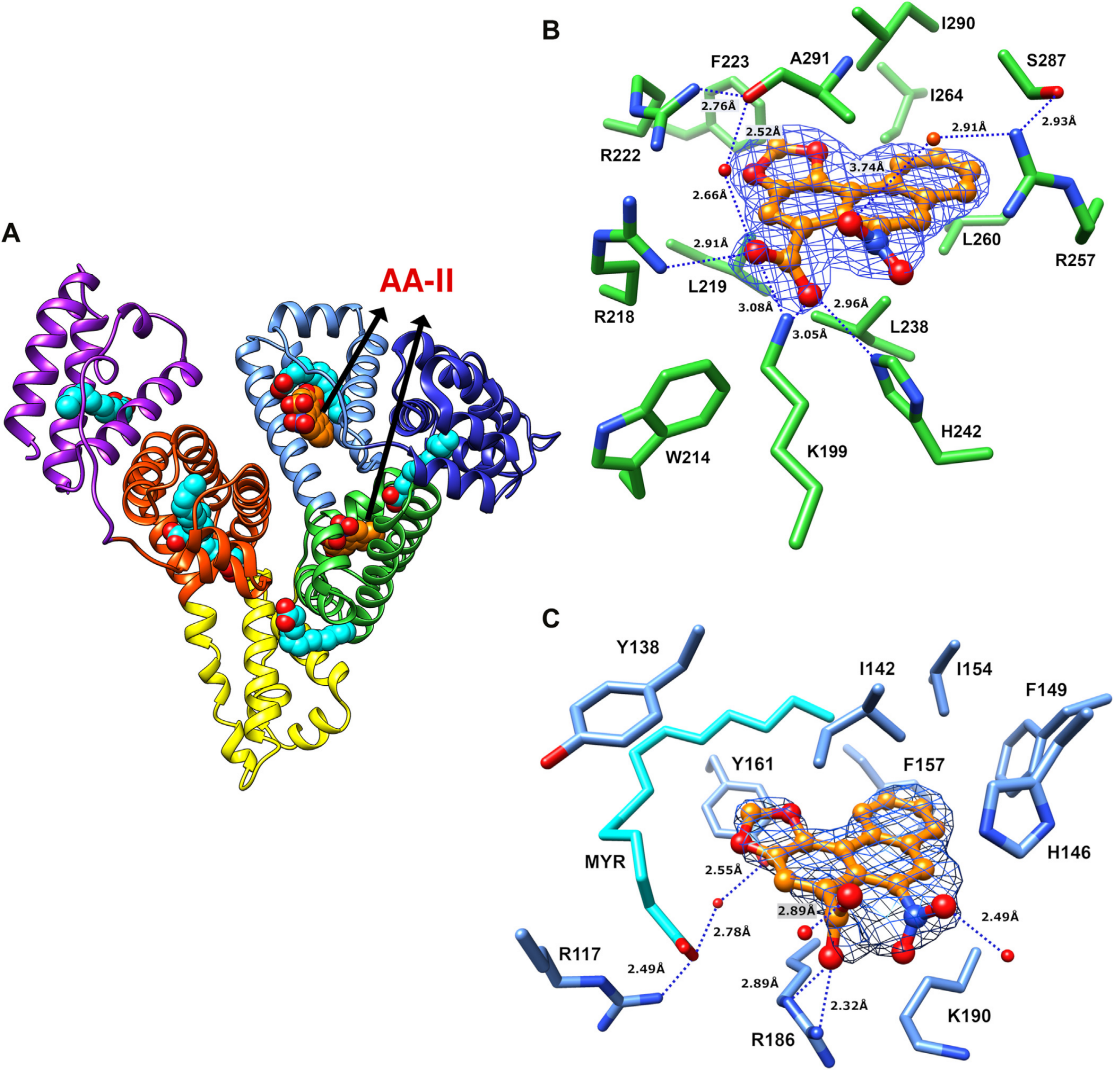
**Structure of HSA**_**MYR**_**/AA-II complex.***A*, overall structure of HSA containing AA-II ligands bound to subdomain IIA (FA7; *green*) and at the lip of subdomain IB (FA10; *blue*) adjacent to FA1 (*cyan*). *B*, details of AA-II binding at subdomain IIA depicting I290/A291 above and L238 below the aromatic plane. *C*, detailed view of AA-II binding at subdomain IB in which a myristic acid molecule (*cyan*) occupies FA1 between Y161 and Y138, whereas AA-II is located proximate to H146. The 2*Fo-Fc* electron density maps of AA-II (*blue mesh*) in *B* and *C* are contoured at 1.5σ. Water molecules are depicted as *red spheres* and hydrogen bonding distances as *dotted lines*.

A primary objective of this study is to ascertain the binding characteristics of AA-II and compare these parameters with those of AA-I by crystallizing the HSA_MYR_/AA-II complex and determining its detailed molecular structure. A high-resolution structure of the HSA_MYR_/AA-II complex in Figure 6*A* reveals two locations for the AA-II ligand, namely Drug Sites I (subdomain IIA) and III (subdomain IB). AA-II effectively displaces the corresponding FA7 and FA10 myristate molecules in Drug Sites I and III while leaving FA1 intact. In the following sections, we describe the molecular details of AA-II interactions with Drug Sites I (subdomain IIA) and III (subdomain IB), respectively.

#### Characterization of HSA_MYR_/AA-II interactions within subdomain IIA

The structure of HSA_MYR_/AA-II complexes reveals that AA-II interacts with FA7 at the location originally occupied by a fatty acid molecule as highlighted in Figure 6*A*. This refined conformation appears in the final crystal structure deposited (PDB ID 8RCO) and specific details are presented in Figure 6*B*. Interestingly, the data suggest that two binding orientations are possible in subdomain IIA, namely the carboxylate and nitro groups of AA-II are interchanged within the binding pocket reflecting a 180° flip around the AA-II main axis (refer to Fig. S5, *C* and *D*). Although both orientations have been analyzed and refined, the configuration in which an AA-II carboxylate moiety faces R218 yields the best fit to experimental electron density, resulting in a high average occupancy of 0.99. Nevertheless, it is entirely plausible that AA-II binds at Drug Site I (FA7) in both orientations yet with distinct occupancies.

In the preferred binding orientation (PDB ID 8RCO), the aromatic region of AA-II is bound deeply within the pocket and forms hydrophobic interactions with surrounding residues as illustrated in Figure 6*B*. AA-II interacts with I290/A291 and L238 from above and below the aromatic plane, respectively. L260 and I264 participate in hydrophobic interactions with the furthermost aromatic moiety of AA-II located deep within Drug Site I (refer to Figs. S6*B* and S7, *A* and *B*). Conversely, F223 and L219 surround the methylenedioxy ring yet do not appear to interact strongly with AA-II given their distal location. The carboxylate and nitro moieties of AA-II face toward the entrance of Drug Site I and engage in hydrogen bonding and electrostatic interactions with neighboring residues. K199 forms a salt bridge with the carboxylate group of AA-II, which in turn forms a hydrogen bond with R218 and a water molecule. This water molecule interacts with R222 and the main chain carbonyl oxygen of A291, forming a tight network of interactions above the AA-II general plane (Figs. 6*B* and S6*B*). Additionally, the carboxylate group of AA-II forms a hydrogen bond with H242 while the nitro group extends towards this residue forming a hydrogen bond with a water molecule bound to R257.

#### Characterization of HSA_MYR_/AA-II interactions within subdomain IB

Analogous to AA-I interactions within the HSA_MYR_/AA-I complex, AA-II is located in subdomain IB as depicted in Figure 6*C* albeit at a significantly distinct orientation and spatial configuration when compared to AA-I (refer to Fig. 5*B*). Moreover, in contrast with the HSA_MYR_/AA-I complex, a myristate molecule (FA1) co-occupies the subdomain IB pocket thereby resembling lipid interactions observed in the ligand-free HSA_MYR_ structure (refer to Fig. 4). Difference in electron density at the outer “lip” of this pocket reflects the presence of AA-II, exhibiting a binding mode that is somewhat reminiscent of diclofenac or JMS-053 interactions with subdomain IB (72, 73). While the space between Y138 and Y161 in the HSA_MYR_/AA-I complex is occupied by an AA ligand that replaces the myristate molecule at FA1, AA-II populates the subdomain IB pocket in what appears to be a secondary binding site (Fig. S8, *A* and *B*) by displacing a myristate molecule (FA10). The aromatic moiety of AA-II is shielded from solvent inside the hydrophobic portion of this cleft where it interacts with surrounding residues. F157 forms a T-shaped stacking interaction with the AA-II terminal aromatic ring, while F149 and L154 participate in hydrophobic interactions from a direction comparable to that of F157 (refer to Fig. 6*C*). The I142 residue appears to be involved in a hydrophobic interaction with AA-II from above the molecular plane.

At the newly designated FA10 site, both the carboxylate and nitro groups of AA-II protrude from this pocket towards the solvent, whereby the carboxylate moiety forms hydrogen bonds with the R186 side chain and a water molecule. The nitro group is hydrogen bonded to H146 and a water molecule (Fig. S6*C*). Interestingly, one of the HSA chains in the current crystallographic structure presents an alternate bonding pattern that differs by a water molecule bridging the carboxylate and nitro groups as well as a hydrogen bond from the latter to K190. This structural feature suggests that AA-II interactions at the lip of subdomain IB (FA10) may fluctuate between several possible configurations. Despite the binding of AA-II, the hydrogen bonding pattern between R117, FA1, and Y161 remains undisturbed, thereby resembling ligand-free HSA_MYR_ as myristate and AA-II bind concurrently at FA1 and FA10, respectively (Fig. S8*B*). However, it appears that the refined average AA-II binding occupancy of 0.8 at subdomain IB is lower than that of subdomain IIA, perhaps owing to orientation of the ligand and its relative proximity to solvent. Moreover, the reduced interaction network compared to AA-I is consistent with the proposition that AA-II exhibits a lower binding affinity to HSA subdomain IB.

Collectively, the structural data suggest a common binding mode for AA-I and AA-II to HSA in which the aromatic regions of these AA ligands form extensive hydrophobic interactions with the lipophilic sections of HSA binding sites, whereas the carboxylate and nitro groups engage in favorable interactions, primarily with basic residues that surround the binding pockets. These interactions are supplemented and strengthened by a robust network of hydrogen-bonded water molecules at the entrance to each pocket. Another characteristic feature defining these AA-I and AA-II interactions is that the carboxylate moiety of AA compounds adopts an orientation similar to the corresponding carboxylate group of myristate molecules in ligand-free HSA_MYR_ (refer to Fig. S3). This finding underscores the importance of such a binding configuration and reinforces the suggestion of an underlying motif governing the binding mode of AA-I and AA-II to HSA. Specifically, AA-I in subdomain IB (FA1) and AA-II in subdomain IIA (FA7) resemble the orientations of myristic acids that previously occupied these sites. At all the binding interfaces between HSA and AA species, the ligands are bound tightly *via* a relatively dense network of interactions and deeply enveloped within their respective binding pockets. As a case in point, AA-I binding to site FA1 in subdomain IB is characterized by ∼467 out of a total 487 Å^2^ (∼94–98%) in buried solvent-accessible surface area (SASA). Similarly, AA-II bound in subdomain IIA (FA7) is exposed to the surrounding solvent by two narrow channels and exhibits comparable surface area burial (∼94%). Even in the case of AA-II bound at the lip of subdomain IB, a position of lower ligand occupancy and greater solvent exposure, the amount of buried SASA remains surprisingly high (∼93%) as noted in Table S3. In summary, the highly specific binding of AA-I and AA-II to HSA_MYR_ results in significant SASA burial and shielding from the surrounding solution.

## Discussion

The primary goal of this study is to characterize the molecular forces governing interactions between aristolochic acids and their activated metabolites with serum albumins. An ancillary objective is to elucidate how these interactions modulate the transport of toxic agents to various organs and tissues while reducing their biodegradation and clearance. Such information is essential and relevant to understanding the metabolic pathways of environmental compounds including their mechanisms of distribution and carcinogenesis. A notable feature of this study is the development of unique approaches that afford high-resolution X-ray structures of human plasma-derived albumins in the absence and presence of ligands. In summary, we report the following novel findings: (i) serum albumins extend the half-life and reactivity of otherwise labile *N*-sulfonyloxyaristolactam I, an activated metabolite of AA-I, presumably expanding its window of toxicity and ability to form DNA adducts; (ii) HSA interactions with AAs and their metabolites are differentially modulated by the presence of fatty acids as deduced *via* fluorescence quenching assays; (iii) ITC profiles monitoring the association of AA-I and AA-II with defatted HSA exhibit two binding sites characterized by micromolar affinities that generally corroborate structural analysis; and, (iv) high resolution structures of HSA_MYR_, HSA_MYR_/AA-I, and HSA_MYR_/AA-II afford identification and structural elucidation of AA interactions within subdomain IB (AA-I and AA-II) and subdomain IIA (AA-II).

### Mechanistic role of HSA in AA sequestration, transport, and disposition

Serum albumin assumes numerous functions in the human body that include ensuring the solubility and transport of long-chain fatty acids in circulation, delivering hormones and other natural substances to tissues, and rendering potential toxins such as environmental compounds less harmful by binding irreversibly and/or transporting these small molecules to disposal sites (50). Therapeutic agents can also be modulated by binding to albumin, as this transport protein often limits the rate of metabolism and excretion of numerous drugs (49). Our interest in serum albumin originated from studies on AAs and their mechanisms of metabolic activation. Exposure to AAs occurs through the ingestion of natural herbal remedies or tainted food products as proposed for endemic areas of aristolochic acid nephropathy in the Balkan countries (74).

Our understanding of AA fate in the human body is limited as studies on their metabolism and disposition derive from studies in rodents (8, 75), cultured cells (40, 41, 46, 76), and recently *via* human liver-kidney organs-on-a-chip (44). The current model for AA disposition suggests that following ingestion and absorption from the intestinal lumen, AAs are delivered through the portal vein to the liver. Evidence reveals that hepatocytes not only detoxify AAs to aristolactams and C7-hydroxyaristolochic acids but produce the mutagenic species *N*-hydroxyaristolactams and *N*-sulfonyloxyaristolactams (36, 44). At this stage, glucuronidated and sulfonated conjugates of lactams and C7-hydroxyaristolochic acids can also be formed, and most are excreted with the bile. Significantly, unmodified AAs and their metabolites can appear in the blood and be transported *via* circulation to multiple organs.

Given the unstable nature of AL-NOSO_3_ as demonstrated in this study and the observation that serum albumins increase the life-span of 1-sulfonyloxymethylpyrene, a carcinogenic metabolite of 1-methylpyrene (77, 78, 79), we suggest that binding of AL-NOSO_3_ in lipophilic regions of the serum albumin pocket(s) might elicit a protective effect. Indeed, our mechanistic studies indicate that the presence of albumins extends t_1/2_ and prolongs the reactivity of AL-I-NOSO_3_ with DNA by at least 20-fold. These experiments support the proposed mode of HSA protective action towards AL-NOSO_3_, indicating that under equilibrium conditions and an excess of HSA at any given time, a percentage of AL-I-NOSO_3_ is present in an unbound form. This assessment is consistent with previous studies employing plasma ultrafiltration following AA ingestion, whereby high albumin concentrations (∼600 μM) significantly exceed the levels achieved by these toxins *in vivo* (46). Since the t_1/2_ of HSA in humans is approximately 14 days and most AA species in the bloodstream are HSA-bound, the latter will significantly prolong overall exposure to these mutagenic compounds following ingestion.

### Characterization of HSA interactions with AA-I and AA-II

Our initial binding assays employing fluorescence quenching measurements of the single HSA Trp^214^ located within subdomain IIA suggest that association of AAs and their metabolites are in the low micromolar range, thereby supporting the notion that at least 90% of these compounds are transported *via* circulation in their HSA-bound form. While our fluorescence quenching assays serve solely as an initial assessment of apparent dissociation constants, the latter have proven enlightening for a comparative analysis of HSA interactions with AA-I, AA-II, and metabolites including evaluating the impact of fatty acids on their relative affinities. Isothermal titration calorimetry (ITC) affords a detailed characterization of AA-HSA interactions and quantitative determination of ligand binding affinities. Application of this technique precludes the need for specific probes, thereby avoiding limitations associated with spectroscopic measurements in terms of optimizing protein:ligand ratios.

The acquisition of calorimetric profiles for AA-HSA interactions reveals that AA-I and AA-II are characterized by two binding events with distinct affinities. While the AA-I binding affinity for site 1 (K_A1_ = 1.5 · 10^6^ M^−1^) is nearly three-fold that of site 2 (K_A2_ = 5.4 · 10^5^ M^−1^), comparable affinities are observed for AA-II binding to sites 1 (K_A1_ = 8.4 · 10^5^ M^−1^) and 2 (K_A2_ = 9.0 · 10^5^ M^−1^). Our analysis of the binding energetics characterizing this complex macromolecular system necessarily requires the acquisition of calorimetric data under various solution conditions (*e.g.*, buffer, pH, temperature) to derive intrinsic thermodynamic parameters and resolve linked processes (80). Nevertheless, the binding profiles reported herein are consistent with the structural data deduced *via* crystallographic analysis that reveals the existence of two distinct binding sites within the HSA macromolecule exhibiting moderate to high affinities. The characteristic binding isotherms observed for AA-I and AA-II interactions with defatted HSA reflect distinct energetic signatures which suggest differences in the primary thermodynamic driving forces governing AA-HSA recognition.

### Structural insights into HSA complexes with AA-I and AA-II

The crystallographic data obtained for AA-I and AA-II binding to HSA at 1.9 Å ranks amongst the seven highest resolution structures available for HSA-ligand complexes, thereby affording detailed molecular analysis at the atomic level in a crystalline environment. An important insight gleaned from the study of HSA_MYR_/AA complexes is that AA-I and AA-II are capable of displacing and replacing medium-chain myristic acid molecules that are weaker serum albumin binders. The notion of AA transport and shielding by HSA is further supported by the high binding-induced surface burial of AAs, which only allow for limited access to hydrophilic groups that are engaged in tight interactions with neighboring residues, thereby reducing the dissociation rates of HSA-bound AAs. The structural data reveal that two molecules of AA-II are bound to HSA, namely one in the pocket of subdomain IIA and another at the lip of subdomain IB. Despite the higher ligand occupancy observed for subdomain IIA relative to IB, these interactions are accompanied by comparable burial of solvent-accessible surface area. While AA-II interacts with both sites in the crystallographic complex, the structure of AA-I is characterized by single-site occupancy in the IB subdomain of HSA_MYR_. The presence of AA-I in subdomain IB corroborates prior studies employing site-specific competitive circular dichroism approaches whereby AA ligands displace biliverdin from its primary site (57).

In contrast with the crystallographic structures, equilibrium binding studies provide evidence that both AA-I and AA-II interact with at least two sites in defatted HSA. Specifically, calorimetric profiles reveal that two molecules of AA-I and AA-II bind HSA with distinct affinities. The disparity observed between solution and *crystallo* measurements may stem from a lower AA-I:HSA ratio achieved in the latter as a consequence of reduced ligand solubility. Although we cannot unequivocally assign a specific binding site for a second AA-I molecule within HSA, our data in conjunction with the body of evidence gleaned from previous studies suggests that this ligand might be accommodated in subdomain IIA analogous to the HSA_MYR_/AA-II complex. These observations suggest that the calorimetrically derived high affinity binding site for AA-I is located in subdomain IB, whereas the low affinity binding site is situated either in subdomain IIA or at an alternate location not yet detected *via* crystallographic analysis.

There are several other plausible scenarios to rationalize the finding that a single AA-I molecule is bound to subdomain IB in crystal structures of the resultant HSA_MYR_/AA-I complex when compared with the two-site binding observed in calorimetric studies employing defatted serum albumin. Specifically, such differences may be enhanced by the absence of fatty acids, thereby avoiding potential competition between AA ligands and myristic acid for available binding sites. Previous reports reveal that myristic acid interacts with numerous binding sites in HSA, exhibiting affinities ranging from 10^4^ to 10^7^ M^−1^ (63). Remarkably, five of these binding events have been associated with significant conformational switches in the HSA molecule. Myristate binding to HSA induces substantial conformational changes, some of which involve rotations of domains I and III relative to domain II.

Myristic acid-induced allosteric effects may either compete for a binding site or enhance interactions of other ligands as documented in the literature (61, 62, 81, 82). Our fluorescence quenching assays are consistent with such findings as AA-HSA affinities are differentially modulated by the presence of fatty acids. Considering the potential interdependence of AA sites within the HSA binding domains, one or more mechanisms may collectively suppress the interactions of AA-I at site IIA irrespective of moderate to high affinities measured for this ligand in solution using defatted HSA These observations require further elaboration yet suggest the possibility that AA-I binding to subdomain IB might impact interactions with subdomain IIA, the latter exacerbated by modulating events such as competition with myristic acid in FA7 as surmised based on inspection of the HSA_MYR_ crystal structure.

It is well established that Sudlow’s Site I (subdomain IIA) is comprised of distinct hydrophobic and hydrophilic regions (83) that may serve as an optimal HSA binding site for AAs and their activated species. Although our crystallographic measurements suggest that AL-I-NOSO_3_ and AL-I-NOH bind in this particular pocket, attempts to obtain higher resolution structures have met with limited success. Consequently, we cannot unequivocally determine whether captured crystals retain the original carcinogens or their decay products. Since albumins apparently preserve the integrity and activity of AL-I-NOSO_3_, it is possible that these structures reflect binding of the original species *in lieu* of decomposed derivatives. It is tempting to speculate that albumin assumes a dual role in the toxicity of aristolochic acids, namely *via* trapping and detoxifying active AA species circulating in free form following heterolytic cleavage and/or preventing the decomposition of activated AA metabolites.

The structures of HSA_MYR_-AA presented in this study reveal that AA-I is bound within subdomain IB in a configuration comparable to hemin, as both ligands are relatively planar and sandwiched between the two key tyrosine residues (Tyr138 and Tyr161), thereby penetrating deeply into the subdomain IB cleft (Fig. S9*A*). Similarly, AA-II is bound within Drug Site I in a manner reminiscent of the warfarin binding mode, another generally planar polycyclic molecule (84) (Fig. S9*B*). Considering the comparable binding modes displayed between AA species and their respective binding site prototype ligands (*i.e.*, hemin and warfarin), it is plausible that AA-HSA interactions induce and are subjected to similar reciprocal allosteric effects. The latter may play a significant role in either minimizing or potentiating deleterious effects, observations that might be explored further as detoxification mechanisms.

### Biological implications

Our experimental strategy has been designed to explore the mechanistic role of HSA in modulating the short- and long-term toxicity of AAs by employing complementary biochemical, biophysical, and structural approaches. Following oral ingestion, absorption, and hepatic processing, AAs and their metabolites are released to the bloodstream and sequestered by serum albumin. The latter assumes a dual role in protecting solvolysis-prone species and restricting clearance/elimination, thereby serving as a reservoir facilitating distribution to multiple organs where these compounds ultimately elicit their carcinogenic and toxic effects. The high affinity observed for AA-HSA interactions in conjunction with the increased half-life of reactive species provide compelling evidence that metabolite transport to the target organs culminating in DNA adduct formation is exquisitely regulated by HSA. Conversely, endogenous and/or exogenous compounds may function as allosteric effectors that modulate HSA binding activity, thereby impacting AA transport and toxicity. Knowledge of such potential mechanisms may assist in advancing therapeutic applications by utilizing the modulatory effect of non-toxic ligands to effectively displace and detoxify active metabolite species. A complete understanding of AA-HSA binding mechanisms is therefore an essential prerequisite for the rational design, development, and implementation of effective therapeutic interventions against AA carcinogenicity and toxicity. Collectively, our findings yield significant insight regarding the complex pathways by which AAs exert their carcinogenic effects, while simultaneously unraveling the potential participation of HSA in curbing and/or exacerbating AA toxicity.

## Experimental procedures

### Caution

AA-I, AA-II, AL-I-NOH, AL-II-NOH, AL-I-NOAc, and AL-I-NOSO_3_ are known or suspected human carcinogens and nephrotoxins that should be handled in a well-ventilated fume hood with protective clothing.

### Chemicals

AA-I and AA-II were purified from *A. indica* by preparative high-performance liquid chromatography (HPLC). AL-I-NOH, AL-II-NOH, AL-I-NOAc, and AL-I-NOSO_3_ were synthesized in-house as described (85). The purity and integrity of these compounds were routinely verified by nuclear magnetic resonance (H^1^-NMR) spectroscopy, HPLC, and liquid chromatography coupled with tandem mass spectrometry (LC-MS/MS). All AA compounds were dissolved in DMSO at concentrations of 20 to 50 mM. AA-I and AA-II standards were aliquoted and stored as single stocks at −20 °C. Active AA metabolites were stored in small aliquots at −80 °C to avoid deterioration *via* freezing/thawing cycles, defrosted prior to each experiment, and discarded following single use.

### Albumin sources and other materials

Bovine serum albumin (BSA, catalog number A2153), deionized water free of nucleases and proteases, salmon sperm DNA (ssDNA), BME, anhydrous butanol, phosphate buffered saline, and sodium myristate were purchased from Sigma Aldrich. Sigma Aldrich also supplied two human serum albumin (HSA) samples derived from pooled human plasma: HSA product numbers A3782 (HSA_A3782_) and A8763 (HSA_A8763_). The HSA_A3782_ preparation is essentially fatty acid and globulin free containing less than 0.005% of fatty acids and <1% of globulins. HSA_A8763_ includes some fatty acids but is essentially free of globulins. All other chemicals were ACS grade. PD-10 Sephadex columns and Amicon Ultra centrifugal filter units (10 kDa cut off, 0.5 ml and 15 ml) were purchased from GE Healthcare and Millipore, respectively.

### Small-scale preparation of mercaptoalbumins

Incorporating a protocol to ensure that the single cysteines in HSA_A3782_, HSA_A8763_ (*i.e.*, CysSH34) and BSA_A2153_ (*i.e.*, CysSH58) retain their unmodified structure, we performed a mild reduction with BME followed by buffer exchange on PD-10 columns. Briefly, the albumin samples (50 mg) were dissolved in 2 ml of 100 mM potassium phosphate buffer (KPi, pH 7.5) and the protein concentration verified by UV spectrophotometry (ε_280_ = 35,495 M^−1^ cm^−1^). It is important to note that we first attempted gel filtration of the original HSA_A3782_ sample on a Superdex 75 column. However, the eluting protein exhibited a large shallow peak suggesting that the majority of this sample is present in the dimeric CyS34-CyS34 form (data not shown). Therefore, albumin solutions prepared as described were incubated with 40 μmol BME (∼50-fold excess) on a slowly rotating platform at room temperature for 90 min. The remaining BME was removed by gel filtration on a PD-10 column according to manufacturer instructions. A typical titration profile of HSA with BME is illustrated in Fig. S10*A*. Following gel filtration, the HSA and BSA monomeric mercaptoalbumin stocks (∼200 μM) were aliquoted and stored at −80 °C. UV spectrophotometry and Ellman’s assay (86) confirmed that at least 97% of HSA and BSA underwent reduction to their respective CySH34 and CySH58 forms. This represents a substantial improvement when compared to the original samples that contained nearly 10% reduced species as illustrated in Fig. S10*B* for HSA_A3782_. Monomeric mercaptoalbumins BSA_A2153_ and HSA_A3782_, hereafter designated as BSA and HSA, are used for the majority of studies presented in this manuscript. When HSA_A8763_ and HSA_A3782_ mercaptoalbumins are employed for fluorometric studies, the source of each sample is indicated accordingly.

### Impact of BSA on AL-I-NOSO3 stability evaluated *via* ESI-liquid chromatography/mass spectrometry

A stock solution of AL-I-NOSO_3_ in DMSO was thawed and immediately diluted to 30 μM in 50 mM KPi (pH 7.5). This compound was incubated in the absence and presence of 600 μM BSA at 37 °C over the following time spans: 0, 15, 30, 60, and 90 min; 24, 48, and 72 h. At the indicated time interval, 100 μl aliquots were mixed with an equal volume of ice-cold methanol and the samples centrifuged for 5 min at 15,000 rpm in a benchtop centrifuge to precipitate the protein. The resultant supernatants were analyzed on a Quattro LC Mass spectrometer (Waters-Micromass) operated in electrospray negative mode equipped with a Hewlett Packard series 1100 HPLC system and an Aquasil C18 1 × 150 mm column. Samples were eluted from the HPLC column into the mass spectrometer in 5 μl of 0.001% ammonia and 1:1 acetonitrile:water with a flow rate of 0.05 ml/min. Multiple reaction monitoring (MRM) mode was set to detect two MS/MS transitions specific to AL-I-NOSO_3_, namely 388.10→308.20 and 388.10→292.50. Peak areas of the resulting MRM mass chromatograms were integrated and the amount of AL-I-NOSO_3_ calculated as a percentage of starting material at time zero.

### Activity of AL-I-NOSO3 with DNA in the absence and presence of HSA

AL-I-NOSO_3_ (20 μM in 20 mM KPi, pH 7.5) was incubated in the absence or presence of 600 μM HSA_A3782_ at 37 °C over the following time intervals: 0, 1, 2, 4, 6, and 22 h. At each time point, 20 μl of AL-I-NOSO_3_ or AL-I-NOSO_3_/HSA sample was withdrawn and added to 180 μl of unmodified ssDNA in 20 mM KPi to achieve a 200 μl solution comprised of 0.5 mg/ml ssDNA, 2 μM AL-I-NOSO_3_, and either 0 or 60 μM HSA. Reactions between AL-I-NOSO_3_ and DNA were allowed to proceed at 37 °C for 30 min and terminated by addition of an equal volume of ethyl acetate followed by vigorous vortexing for 2 min to extract unbound AL-I-NOSO_3_ and its decomposition products. Following centrifugation at 15,000 rpm for 3 min at room temperature, the upper layer containing unreacted lactam derivatives was discarded and subsequent extraction steps with 150 μl ethyl acetate repeated twice. Each reaction was performed in triplicate and the resultant samples stored at −80 °C until extraction of HSA and DNA precipitation. The frozen samples were centrifuged under vacuum for 5 to 10 min to ensure evaporation of the remaining ethyl acetate. HSA was removed by three consecutive rounds of extraction using equal volumes of phenol-chloroform-isoamyl alcohol. Irrespective of the absence or presence of HSA, all samples were processed in a comparable manner. DNA from the upper aqueous phase was precipitated by a standard ethanol-sodium acetate protocol. Pelleted DNA was dissolved in 300 μl of water and its concentration determined by measuring the UV absorption at 260 nm (1 OD_260_ corresponding to 50 μg/ml) on a Nanodrop instrument. Samples were stored at −80 °C until adduct analysis *via* ^32^P-postlabeling as described below.

### DNA adduct analysis by ^32^P-postlabeling

Over the past several years, protocols for DNA digestion and sources of enzymes for adduct analysis have been modified from our initial reports. A detailed description of the digestion procedure is provided in this section. Enzymes were obtained from the following sources: Micrococcal nuclease (*Staphylococcus aureus*) and potato apyrase were purchased from Sigma Aldrich; Bovine spleen phosphodiesterase II and nuclease P1 (*Penicillium citrinum*) were purchased from MP Biomedicals; and polynucleotide kinase lacking 3′-phosphatase activity was purchased from New England Biolabs. Gamma-^32^P-ATP (6000 Ci/mmol; 10 μCi/μl) was obtained from PerkinElmer. DNA (5 μg) was digested in a 100 μl mixture containing 20 mM sodium succinate (pH 6.0), 8 mM CaCl_2_, 0.8 units of micrococcal nuclease, and 0.04 units of phosphodiesterase II. Reactions were allowed to proceed overnight at 37 °C. Following digestion, 1 μl of 100 mM zinc chloride and 1 unit of nuclease P1 were added, and the mixtures incubated for 1 h at 37 °C. For standard curves, synthetic oligonucleotides containing a single dA-AL-II or dG-AL-II in the center of their 24-mer sequences were digested as a mixture of 15, 30, and 60 fmol and processed in parallel with DNA samples (35). Digestion mixtures of synthetic standards also included 5 μg of unmodified ssDNA and the remaining steps of the procedure were conducted as described (35). Briefly, adducted 3′-phosphate nucleosides were enriched by two rounds of butanol extraction, dried under vacuum, and 5′-labelled in the presence of gamma-^32^P-ATP and polynucleotide kinase. The labeled samples were resolved by PAGE. Gels were exposed on a phosphor screen (GE Healthcare) for 75 min and overnight. Results were visualized employing a Typhoon system and densitometry conducted using Image QuaNT v*5.2* (Molecular Dynamics). The AL-DNA adduct levels (as a sum of dG-AL-I and dA-AL-I) are plotted against the incubation times of AL-I-NOSO_3_ prior to DNA addition. AL-DNA levels are presented as mean values and standard deviations in Sigma Plot v13.0 (SPSS Inc) generated graphs.

### Steady state HSA fluorescence studies

Equilibrium fluorescence spectra of monomeric HSA samples were collected on an ISS PC1 photon counting spectrophotometer. An excitation wavelength of 295 nm was employed for HSA_A3782_ and HSA_A8763_ to selectively monitor tryptophan and avoid contributions of tyrosine residues. The excitation and emission slits were set to 1 nm and 2 nm, respectively. Prior to initiating the experiment, a stock solution of each AA compound (*i.e.*, AA-I, AA-II, AL-I-NOH, AL-II-NOH, AL-I-NOAc. and AL-I-NOSO_3_) was prepared in DMSO by serial dilution to a 100-fold excess of their final concentration employed in the quenching experiments. HSA samples were diluted to 0.5, 1.0, and 2.5 μM in 10 mM KPi buffer (pH 7.5) and divided into 1 ml aliquots in Eppendorf tubes. A 10 μl aliquot of each AA compound was added to the HSA solutions to achieve a 0.2 to 50 μM concentration range in 1% DMSO. Each AA-HSA mixture was allowed to equilibrate at room temperature for at least 5 min, transferred to a 10 mm × 5 mm quartz cuvette, and emission spectrum recorded over the wavelength range of 310 to 460 nm employing a 2 nm step interval. Following each measurement, the quartz cuvette was cleaned, and the experiment repeated with the next AA:HSA ratio. Reference emission spectra acquired for HSA in the absence and presence of 1% DMSO confirmed that this co-solvent does not appreciably alter the fluorescence signal. Moreover, the emission spectra for each standard solution of AA compound studied revealed a negligible fluorescence signal in the 320 to 440 nm range at concentrations below 25 μM. Peak emission intensities at 340 nm were employed to construct fluorescence titration profiles for each AA compound.

### Analysis of fluorescence quenching assays

Fluorescence titration profiles of the HSA quenching assays were generated using SigmaPlot 13.0. When applicable, the mean and standard deviations obtained from 3 to 5 independent measurements are reported. In order to compare HSA preparations across AA ligands, we employed a nonlinear equation that does not specifically consider multiple site binding yet provides significant insight regarding the relative differential binding properties of various AA ligands. Invoking the SigmaPlot software suite, we applied the following binding model (87):

$$F=F_{\text{free}}-\left(F_{\text{free}}-F_{\text{bound}}\right)*\left(\frac{\left(Pt\;+\;Lt\;+\;\text{KD}\right)\;-\;\sqrt{{\left(\;-\left(Pt\;+\;Lt\;+\;KD\right)\right)}^{2}\;-\;4P_{t}L_{t}}}{2}\right)/Pt$$

where F represents the fluorescence signal observed at each ligand concentration (L_t_) and P_t_ is the total protein concentration. Upon fitting, one obtains values of F_free_ and F_bound_ that correspond to the fluorescence of unbound and fully bound HSA, respectively. The number of binding sites and their respective affinities has been determined for AA-I and AA-II interactions with HSA *via* application of isothermal titration calorimetry as described in the following section.

### AA-HSA interactions characterized *via* isothermal titration calorimetry

Binding parameters for the association of defatted HSA_A3782_ with AA-I and AA-II were determined calorimetrically employing a MicroCal VP-ITC instrument (Malvern Panalytical). Protein stock solutions (0.5–1.5 mM) were prepared by BME reduction to disrupt potential CyS34-CyS34 dimers and dialyzed against three 500 ml exchanges of 10 mM sodium phosphate buffer (pH 7.5). Aliquots of the respective AA ligand stocks were dissolved in protein dialysate and the final DMSO concentration adjusted to 2%. The HSA standard was prepared by diluting an appropriate aliquot in dialysate containing 2% DMSO to match the syringe solution contents. Each ITC experiment consists of thirty consecutive 10.0 μl injections during which the reaction heat is monitored and integrated over a 5-min interval under constant stirring conditions. The experimental protocol has been designed to ensure that there is at least a 15-fold excess of either AA-I or AA-II (75–200 μM) in the titration syringe relative to the initial HSA concentration (5–10 μM) in the reaction cell.

Binding isotherms acquired at 25 °C were generated by recording the integrated reaction heats normalized for AA concentration *versus* ligand:protein ratio. Analysis of the AA-HSA isotherms employing single, two-site, and sequential binding models in the ORIGIN software exhibited inferior fits as a consequence of difficulties encountered in baseline assignments. We subsequently applied the NITPIC/SEDPHAT/GUSSI suite of software programs (60, 88) to ensure unbiased baseline assignments and peak integrations that facilitated selection of an appropriate binding model to characterize this protein-ligand system. The SEDPHAT program affords significant flexibility by allowing implicit and explicit uncertainty corrections that facilitate assessment of incompetent binding species in the protein and/or ligand. A nonlinear least squares analysis of the resultant profiles invoking a symmetric two-site binding model yields an optimal fit of the isotherms and requisite macroscopic parameters for AA-HSA interactions.

### Large-scale preparation of monomeric HSA and HSA_MYR_ for crystallographic studies

The large-scale procedure employed for the preparation of monomeric HSA differs somewhat from that used to obtain samples on a smaller scale. This is a novel method that appears critical to our success in affording the acquisition of high-resolution HSA crystals and specific details of the procedure are described in this section. HSA_A3782_ was used to prepare monomeric HSA enriched with myristic acid (HSA_MYR_) for all crystallization studies. The protocol to obtain monomeric HSA involved dissolving lyophilized protein at a concentration of 620 μM in 55 ml of 20 mM KPi pH 7.5 (buffer A). The sample was reduced in the presence of 35 mM BME as described above for small-scale preparations. The reducing reagent was removed and concentrated HSA obtained by dividing the protein sample between six Amicon Ultra-15 centrifugal filter units (∼10 ml per tube) followed by centrifugation at 4000*g* for 30 min at 4 °C. Following the first spin, eluent was discarded and sample volume above the membrane reduced to 1.5 ml. A 10 ml volume of buffer A was added followed by another round of centrifugation and this procedure repeated twice. In total, three buffer exchanges were performed yielding six protein solutions of 1.5 ml apiece. These samples were combined and adjusted to the total volume of 15 ml in buffer A. The resultant HSA stock (110 mg/ml) consists of at least 99.8% monomeric protein estimated by UV spectrophotometry and Ellman’s assay. The HSA stock was stored as one 8 ml and 13 × 500 μl aliquots at −80 °C prior to crystallization or preparation of complexes with sodium myristate as described below.

Sodium myristate solution (2.63 mM) was prepared in buffer A, stored at 4 °C until use, and heated at 50 °C in a water bath to allow dispersion of the fatty acid immediately prior to preparation of HSA_MYR_. The myristate solution (62 ml) was quickly cooled and combined with 8 ml of monomeric HSA_A3782_, yielding 189 μM HSA and 2330 μM myristate (12:1 fatty acid:protein ratio). The sample was incubated at room temperature for 1 h with occasional gentle mixing. Excess undissolved myristate was pelleted by two rounds of centrifugation for 5 min each at 4 °C and 12,000*g*. The remaining sample was split between six filter units (Amicon Ultra-15, 10 kDa cut off) followed by centrifugation and three successive buffer exchanges using 0.1 mM sodium myristate in buffer A as described above. This procedure yielded 5.8 ml of 191 mg/ml or 2875 μM HSA enriched with myristic acid (HSA_MYR_) that was stored at −80 °C prior to crystallization studies.

### Crystallization of HSA_MYR_ in the absence and presence of AA-I/II

Solutions of HSA_MYR_/AA-I and HSA_MYR_/AA-II were prepared as follows. Immediately prior to crystallization, the concentration of HSA_MYR_ was adjusted to 140 mg/ml in buffer A containing 0.1 mM sodium myristate. AA-I was suspended in 500 μl of buffer A to achieve a maximal (w/v) concentration of 27 mM. The HSA_MYR_/AA-I mixture was prepared by combining 90 μl of HSA_MYR_ (2.1 mM) and 10 μl of AA-I suspension, yielding 2.7 mM AA-I and 1.9 mM HSA_MYR_ (1.4:1 AA-I:HSA molar ratio). As a consequence of limited AAI solubility, we were unable to achieve higher AA-I:HSA molar ratios. The HSA_MYR_/AA-II solution was formulated in a comparable manner by combining 80 μl of HSA_MYR_ and 20 μl of 26 mM AA-II, resulting in 5.2 mM AA-II and 1.68 mM HSA_MYR_ (3:1 AA-II:HSA molar ratio). Following incubation for 24 h at 25 °C, the HSA_MYR_AA-I/AA-II suspensions were centrifuged to discard undissolved AA-I/II and sodium myristate.

The HSA_MYR_, HSA_MYR_/AA-I, and HSA_MYR_/AA-II solutions were set up for crystallization using the hanging-drop method starting from previously determined conditions (69) and further optimized for each complex. The HSA_MYR,_ HSA_MYR_/AA-I, and HSA_MYR_/AA-II crystals were grown from a condition containing 28 to 30% polyethylene glycol 3350 (PEG 3350) and 25 mM sodium phosphate (pH 7.0). For the HSA_MYR_AA-I and HSA_MYR_AA-II complexes, crystallization drops were prepared by mixing 1 μl of crystallization solution with 1 to 3 μl of a corresponding HSA_MYR_/AA mixture and allowed to equilibrate over a 500 μl reservoir for 24 h at 25 °C. Following equilibration, drops were streak-seeded using a dilute (1:10^3^) solution prepared with the SeedBead kit (Hampton Research) from HSA_MYR_ crystals grown similarly under a crystallization condition containing 31% PEG 3350 and 50 mM sodium phosphate (pH 7.0). Following seeding, crystals appeared within several hours and achieved maximal size in 3 days.

HSA_MYR,_ HSA_MYR_/AA-I, and HSA_MYR_/AA-II crystals appeared as elongated transparent rectangles (Fig. S11*A*) characterized by sharp edges and typical dimensions with the largest crystals reaching approximately 0.2 × 0.2 × 0.5 mm. While the HSA_MYR_ crystals were generally clear and colorless, crystals of the HSA_MYR_/AA-I and HSA_MYR_/AA-II complexes possessed a distinct yellow color indicating the presence of AA-I and AA-II, respectively. Prior to data collection, the crystals were cryo-protected by brief soaking in a reservoir solution supplemented with 10% ethylene glycol.

### Crystallization of HSA with AL-I-NOH and AL-I-NOSO_3_

Since AL-I-NOSO_3_ has a limited t_1/2_ in aqueous solutions, we attempted to utilize the protective effect afforded by HSA binding to obtain HSA-metabolite co-crystallization complexes. Due to the limited solubility of AL-I-NOH and AL-I-NOSO_3_ in buffer A, metabolite complexes with HSA_MYR_ or HSA_DEFATTED_ (HSA_A3782_ mercaptoalbumin prior to complexation with myristic acid) were prepared in a somewhat different manner than that used for AA-I and AA-II. In general, we followed a protocol reported previously (54) incorporating several additional modifications. Stock solutions of AL-I-NOH and AL-I-NOSO_3_ were dissolved in DMSO at concentrations of 55 mM and 52 mM, respectively. Appropriate aliquots of these stock solutions were immediately added to HSA_DEFATTED_ at a 3 to 5 fold excess with the caveat that total DMSO concentrations did not exceed 15%. The solutions of HSA_DEFATTED_/AL-I-NOH and HSA_/DEFATTED_/AL-I-NOSO_3_ were incubated overnight and washed repeatedly with buffer A using an Amicon centrifugal filter device with a 10 kD cutoff. The resultant complex solutions retained a strong orange color, confirming the presence of AA metabolites. These solutions were used for crystallization of HSA_DEFATTED_/AL-I-NOH and HSA_DEFATTED_/AL-I-NOSO_3_ under conditions reported previously for defatted HSA (89). Both HSA complexes yielded optimal crystals by vapor diffusion using 33% PEG 3350, 25 mM sodium phosphate, and 1:1 complex/reservoir solution (volume ratio). Drops were streak-seeded within 24 h using unliganded HSA_DEFATTED_ crystal seed stock. Following seeding, crystals appeared within 2 to 3 days and grew to maximal size in 7 days.

Complexes of HSA_MYR_/AL-I-NOH and HSA_MYR_/AL-I-NOSO_3_ were prepared in an analogous manner employing myristate-supplemented buffer A for the washing step. The resultant solutions possessed a slightly lighter yellow color than those of the HSA_DEFATTED_ complexes and were used for crystallization experiments as described in the previous section for HSA_MYR._ It is relevant to note that small orange crystals often formed within the crystallization drops (Fig. S11, *B* and *C*) presumably reflecting unbound AL-I-NOH/NOSO_3_. Apparently, specific crystallization conditions such as high PEG concentration may decrease the solubility of AA metabolites or force HSA into a conformation where these species bind less favorably, resulting in partial dissociation of the HSA/AL-I-NO-complexes and potential generation of degradation products. This effect is more pronounced for HSA_MYR/_AL-I-NOH/NOSO_3_ than for defatted HSA. Crystals obtained in this manner exhibited reasonable diffraction yielding structures nearly identical to those of unliganded HSA_MYR_ with almost no significant difference electron density peaks.

In the case of HSA_DEFATTED_/AL-I-NOH/NOSO_3_, the resulting crystals did not produce high-quality diffraction. Nevertheless, several of these crystals were subjected to X-ray synchrotron radiation, and their structures determined albeit at a rather low resolution. Despite multiple attempts employing different experimental conditions, the resultant HSA_DEFATTED_/AL-I-NOH and HSA_DEFATTED_/AL-I-NOSO3 crystals diffracted relatively poorly as reflected in overall quality and resolution. As a consequence, the resultant crystallographic electron density maps provide only limited structural information that is beyond interpretation at the molecular level (90). Nevertheless, several significant difference electron density peaks appeared in the vicinity of Sudlow’s Drug Site I (subdomain IIA), alluding to the potential presence of a bound HSA-AL-I-NOH/NOSO_3_ metabolite at or near this site (data not shown).

### Data collection and refinement of HSA_MYR_ and the HSA_MYR_/AA-I and HSA_MYR_/AA-II complexes

X-ray diffraction data for the HSA_MYR_ crystals were collected at the I03 beamline, Diamond synchrotron facility (UK), as a part of the second CCP4-BGU course. Data for HSA_MYR_/AA-I was collected at the BM30 beamline of the European Synchrotron Radiation Facility (ESRF) and for HSA_MYR_/AA-II at the P13 beamline, EMBL/DESY. All diffraction images were obtained at a temperature of 100 K. The HSA_MYR_/AA-I diffraction data were integrated with XDS (91) whereas those for HSA_MYR_ and HSA_MYR_/AA-II with DIALS (92). All data sets were scaled and merged by the aimless program within the CCP4i2 software package (93). The structures were solved by molecular replacement (MR) using the PHASER program (94) as part of the PHENIX crystallographic software suite (95). The HSA_MYR_/AA-I complex structure was determined first; phases were resolved by MR using both HSA protein chains included in the PDB entry 5IFO as a search model. Thereafter, the remaining structures were solved by MR, using both protein chains of the HSA_MYR_/AA-I final structure (PDB ID 8RCP) as a search model. The models were refined with the phenix.refine and Refmac (96) programs, coupled with manual building using Coot (97). Myristate and ethylene glycol ligands were manually fitted into their corresponding electron density. The bound AA-I and AA-II molecules could be unequivocally identified and easily fitted into the pronounced difference electron density peaks. The GRADE web server (http://grade.globalphasing.org/) was used to generate ligand restraints for AA-I (PDB CODE: GOQ), AA-II (PDB CODE: GOR), myristic acid (PDB CODE: MYR), and ethylene glycol (PDB CODE: EDO). Major parameters for data collection and refinement of the four structures presented in this study are summarized in Table 2 while the corresponding B-factors and occupancies for each ligand are listed in Table S2. These parameters represent only one specific crystal/structure for each of the complexes discussed, although at least two more crystals have been fully examined, collectively resulting in nearly identical final structures and conclusions.

### Structure analysis and Figure generation

Structures and ligand interactions, as well as hydrogen bonding distances, were analyzed by both the PLIP web server (98) and UCSF Chimera 1.15 (99). The Chimera surfacing tool was used for calculating SASA parameters. Macromolecular 3D structure images were rendered in Chimera and their figures were assembled using GIMP 2.10.20.

### Accession numbers

Atomic coordinates and structure factors for the reported crystal structures have been deposited with the Protein Data bank under accession numbers 8RCO, 8RCP, 8RGK, and 8RGL.

## Data availability

All experimental data are included in the article and/or Supporting Information.

## Supporting information

This article contains supporting information.

## Conflict of interest

The authors declare that they have no conflicts of interest with the contents of this article.

## Supporting information

Figures S1–S11 and Tales S1–S3
