# Supplementary material for: Structural and mechanistic insights into the transport of aristolochic acids and their active metabolites by human serum albumin
Source: J Biol Chem. 2024 May 22;300(7):107358. doi: 10.1016/j.jbc.2024.107358 (PMC11253539; doi:10.1016/j.jbc.2024.107358)
Supplement: Figures S1–S11 and Tales S1–S3 [file mmc1.pdf]

Supporting Information

Structural and Mechanistic Insights into the Transport of Aristolochic Acids and their Active Metabolites by Human Serum Albumin

Sergei Pomyalov, Conceição A. Minetti, David P. Remeta, Radha Bonala, Francis Johnson, Irina Zaitseva, Charles Iden, Urszula Golebiewska, Kenneth J. Breslauer, Gil Shoham, Viktoriya S. Sidorenko, and Arthur P. Grollman

Table S1. Apparent Dissociation Constants (K_{Dapp}) Deduced via Fluorescence Quenching Assays of HSA by Aristolochic Acids and Metabolites …………… S1

Table S2. Ligand B-factors and Occupancies …… S2

Table S3. Solvent Accessible Surface Area of AA Species Buried Upon Binding to HSA …… S3

Figure S1. HSA-AA Binding Profiles Deduced via Fluorescence Quenching Assays of Trp^{214} …… S4

Figure S2. Apparent Dissociation Constants for AA and Metabolite Complexes in the Absence (HSA_{A3782}) and Presence (HSA_{A8763}) of Fatty Acids …… S5

Figure S3. Structure of the HSA_{MYR} Subdomain IB Binding Site and Corresponding Key Amino Acids …… S6

Figure S4. Aristolochic Acid I Bound in the HSA Subdomain IB Site …… S7

Figure S5. *Fo-Fc* and *2Fo-Fc* Electron Density Maps of AA-I and AA-II Bound in Subdomains IB and IIA, respectively …… S8

Figure S6. Ligplot Diagrams Depicting Binding Sites in HSA/AA-I (A) and HSA/AA-II (B, C) …… S9

Figure S7. Aristolochic Acid II Bound to HSA Subdomain IIA (Drug Site I) …… S10

Figure S8. Aristolochic Acid II and FA1 Myristate Bound in HSA Subdomain IB …… S11

Figure S9. Superimposition of Aristolochic Acids I (A) and II (B) with Ligands that Commonly Bind to HSA Subdomains IB and IIA …… S12

Figure S10. Preparation of BME-reduced CySH34 HSA_{A3782} Stock …… S13

Figure S11. Crystals of HSA, Aristolochic Acids, and HSA/AA Complexes …… S14

Table S1. Apparent Dissociation Constants (K_{Dapp}) Deduced via Fluorescence Quenching Assays of HSA by Aristolochic Acids and Metabolites.

Ligand	K_{Dapp} (HSA_{A3782})	K_{Dapp} (HSA_{A8763})
**AA-I**	**0.43 ± 0.03**	**0.43 ± 0.03**
**AL-I-NOH**	0.65 ± 0.06 **	0.74 ± 0.06 **
**AL-I-NOSO3**	0.62 ± 0.10 *	0.45 ± 0.07
**AL-I-NOAc**	0.67 ± 0.13 **	0.59 ± 0.07
**AA-II**	**0.63 ± 0.10**	**0.36 ± 0.03**
**AA-II-NOH**	0.38 ± 0.05 **	0.61 ± 0.05 **

Apparent Dissociation Constants ($\mathbf{K_{Dapp}}$) are reported as the mean ± SD for 3 to 5 independent measurements of AA species in the absence (HSA_{A3782}) and presence (HSA_{A8763}) of fatty acids. Binding data are evaluated via One-Way ANOVA and Tukey Tests employing confidence levels of $p < 0.10$ (*) and $p < 0.05$ (**) to deduce statistically significant differences in $\mathbf{K_{Dapp}}$ for each metabolite relative to the corresponding unmodified AA-I / AA-II ligand.

Table S2. Ligand B-factors and Occupancies.

	HSA-Myr (8RCP)				HSA-AA-I (8RGK)				HSA-AAI (Two Copies) 8RGL				HSA-AA-II (8RCO)				All Structures	
	Chain A		Chain B		Chain A		Chain B		Chain A		Chain B		Chain A		Chain B			
**Ligand**	B-factor	Occupancy	B-factor	Occupancy	B-factor	Occupancy	B-factor	Occupancy	B-factor	Occupancy	B-factor	Occupancy	B-factor	Occupancy	B-factor	Occupancy	Average B-factor	Average Occupancy
**FA1**	51.34	0.92	49.00	0.87	65.94	1	--	--	--	--	--	--	48.38	0.79	49.41	0.81	52.81	0.88
**FA2**	53.33	1	50.72	0.97	54.63	0.92	30.13	0.97	29.03	0.9	62.25	0.95	52.58	0.92	49.97	0.85	47.83	0.94
**FA3**	44.48	0.94	51.61	0.96	50.25	0.89	32.93	0.83	26.69	0.84	48.58	0.86	52.84	1	48.02	1	44.42	0.92
**FA4**	54.50	0.91	56.11	0.93	54.15	0.78	42.91	1	42.26	0.98	54.90	0.85	55.41	0.87	56.20	0.87	52.05	0.90
**FA5**	53.24	1	55.24	0.98	65.21	0.93	51.46	1	53.21	0.96	71.15	1	56.14	0.9	59.48	0.9	58.14	0.96
**FA6**	45.00	0.92	47.17	0.92	55.80	0.77	36.51	0.96	53.21	0.96	62.04	0.79	46.35	0.87	44.69	0.78	48.85	0.87
**FA7**	51.61	0.95	50.87	0.92	64.82	1	39.27	1	34.94	0.93	68.02	1	--	--	--	--	51.59	0.97
**FA10**	49.92	0.82	53.06	0.83	--	--	--	--	--	--	--	--	--	--	--	--	51.49	0.83
**AA-I Site IB**	--	--	--	--	--	--	26.07	0.88	24.94	0.93	68.26	0.91	--	--	--	--	39.76	0.91
**AA-II Site IIA**	--	--	--	--	--	--	--	--	--	--	--	--	39.05	1	37.82	0.97	38.43	0.99
**AA-II Site IB Lip**	--	--	--	--	--	--	--	--	--	--	--	--	47.24	0.79	48.20	0.81	47.72	0.80
**Per-Structure:**	**B-factor**		**Occupancy**		**B-factor**		**Occupancy**		**B-factor**		**Occupancy**		**B-factor**		**Occupancy**			
**Average FA**	51.07		0.93		49.54		0.93		50.52		0.92		51.62		0.88			
**Average AA**					26.07		0.88		46.60		0.92		43.08		0.89			

The table compiles individual B-factors and occupancies of ligands present in the crystal structures. Per-ligand averages appear in the two right most columns while per-chain Fatty Acid and Aristolochic Acid I/II averages are listed in the two bottom lines.

Table S3. Solvent Accessible Surface Area of AA Species Buried Upon Binding to HSA.

Complex	Binding Site	Chain	Total SAS Å^2	Buried SAS Å^2	Buried SAS (%)
**HSA$_{MYR}$/AA-I**	IB	A	487.34	457.22	**93.8**
		B	485.82	478.58	**98.5**
**HSA$_{MYR}$/AA-II**	IIA	A	444.48	416.72	**93.8**
		B	444.80	419.44	**94.3**
	IB (Lip)	A	445.75	415.35	**93.2**
		B	445.93	413.02	**92.6**

Inspection of the HSA$_{MYR}$/AA-I and HSA$_{MYR}$/AA-II complexes reveals that buried solvent accessible surface area exceeds 90 percent for the AA ligands bound to subdomains IB (AA-I/AA-II) and IIA (AA-II).

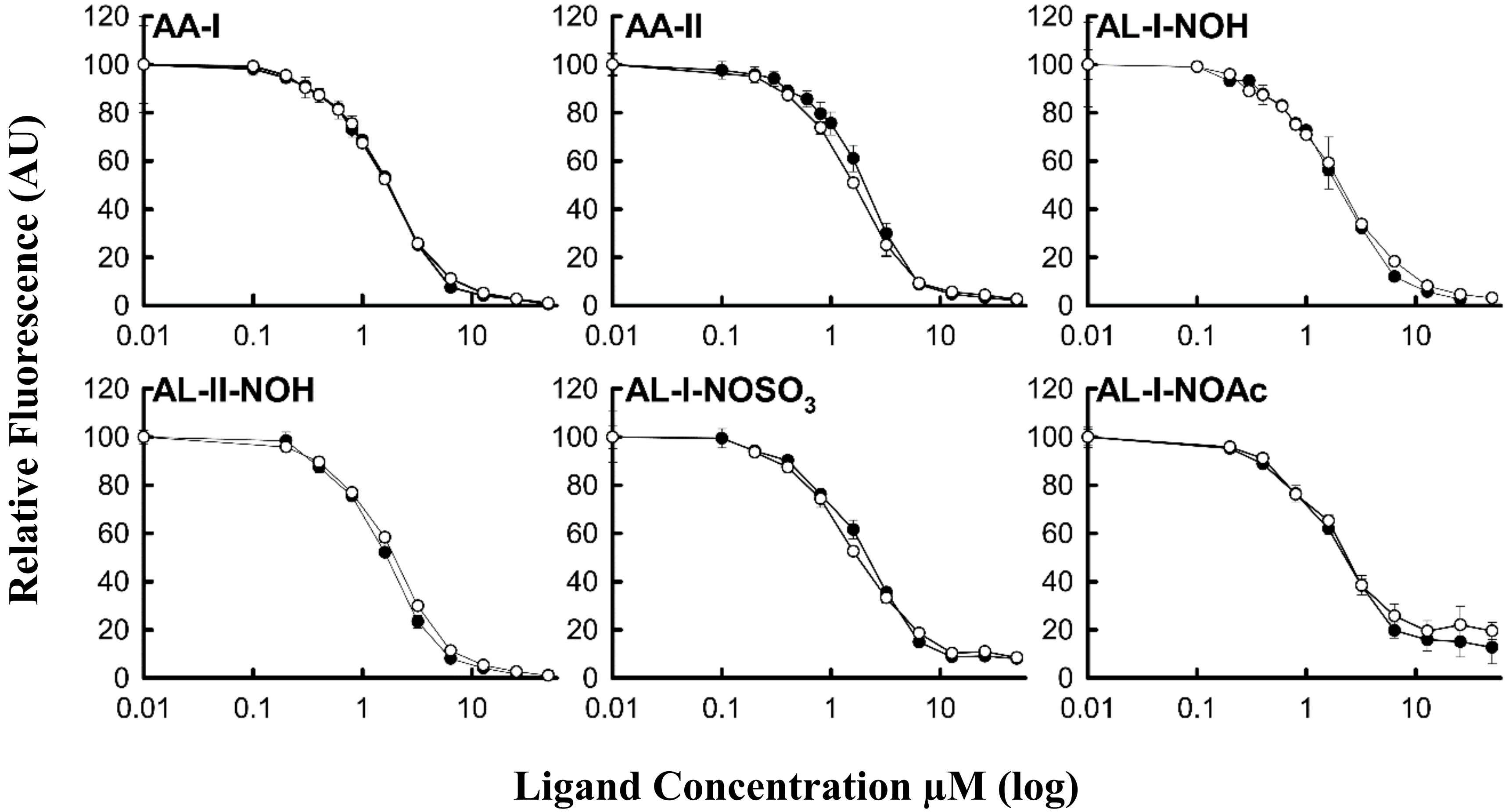

Figure S1. HSA-AA Binding Profiles Deduced via Fluorescence Quenching Assays of Trp[214]. HSA_{A3782} (closed circles) and HSA_{A8763} (open circles) are incubated in the presence of AA-I, AA-II, AL-I-NOH, AL-II-NOH, AL-I-$NOSO_3$, and AL-I-NOAc. The intrinsic Trp[214] fluorescence intensity (λ_{EX} = 295 nm; λ_{EM} = 340 nm) is recorded for each HSA/AA ratio to create the corresponding ligand-induced quenching profile. The maximum fluorescence intensity observed for HSA in the absence of AA species is assigned as reference (100 AU). Each data point represents the *mean* and *standard deviation* for three to five independent equilibrium titration experiments.

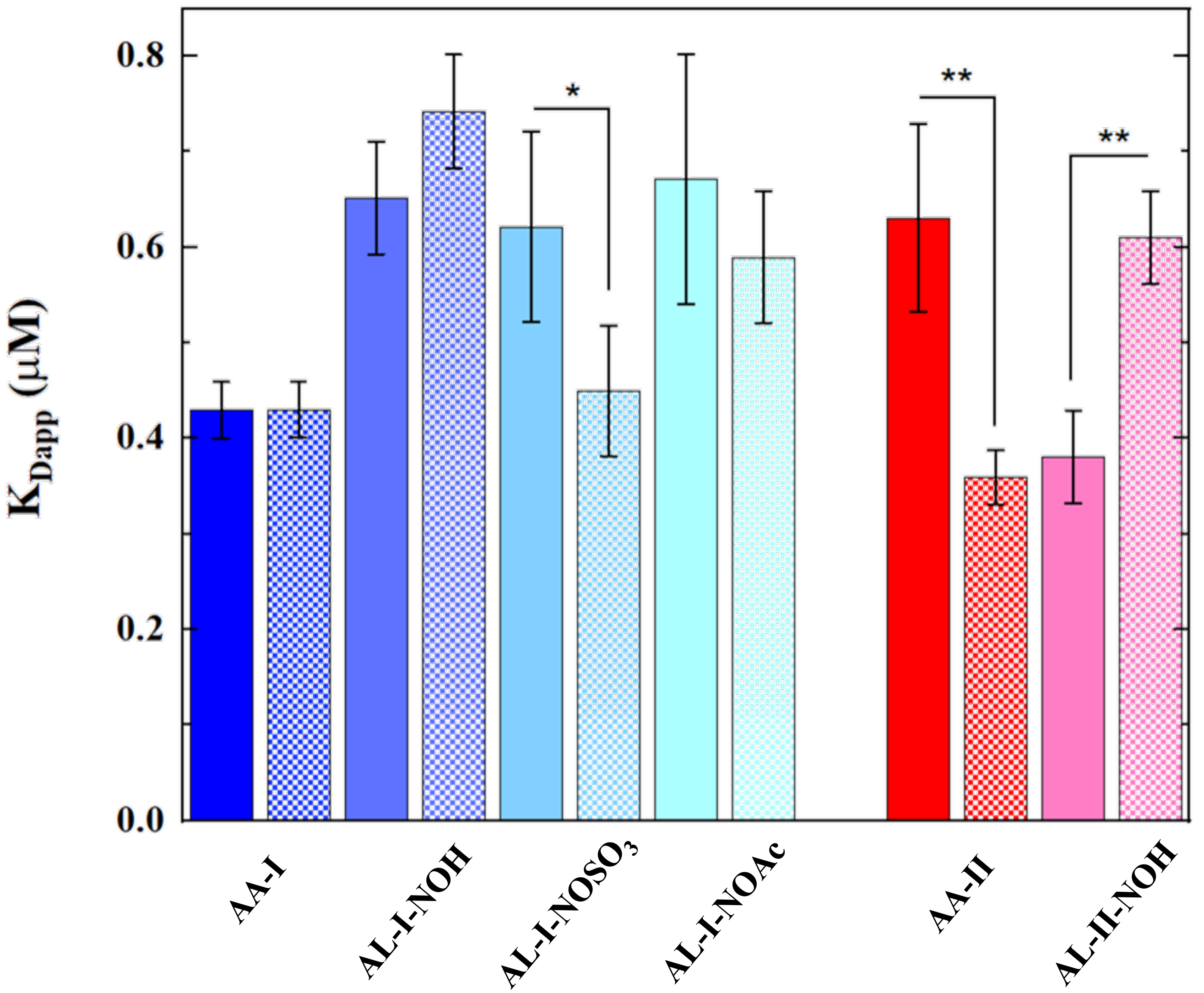

Figure S2. Apparent Dissociation Constants (K_{Dapp}) for AA and Metabolite Complexes in the Absence (HSA_{A3782}) and Presence (HSA_{A8763}) of Fatty Acids. Data are reported as the mean ± SD with statistical significances deduced via One-Way ANOVA and Tukey Tests employing confidence levels of $p < 0.10$ (*) and $p < 0.05$ (**). Respective differences in $\mathbf{K_{Dapp}}$ are calculated for AA ligand interactions with fat-free (HSA_{A3782}, solid) relative to fat-containing (HSA_{A8763}, checkerboard) protein preparations.

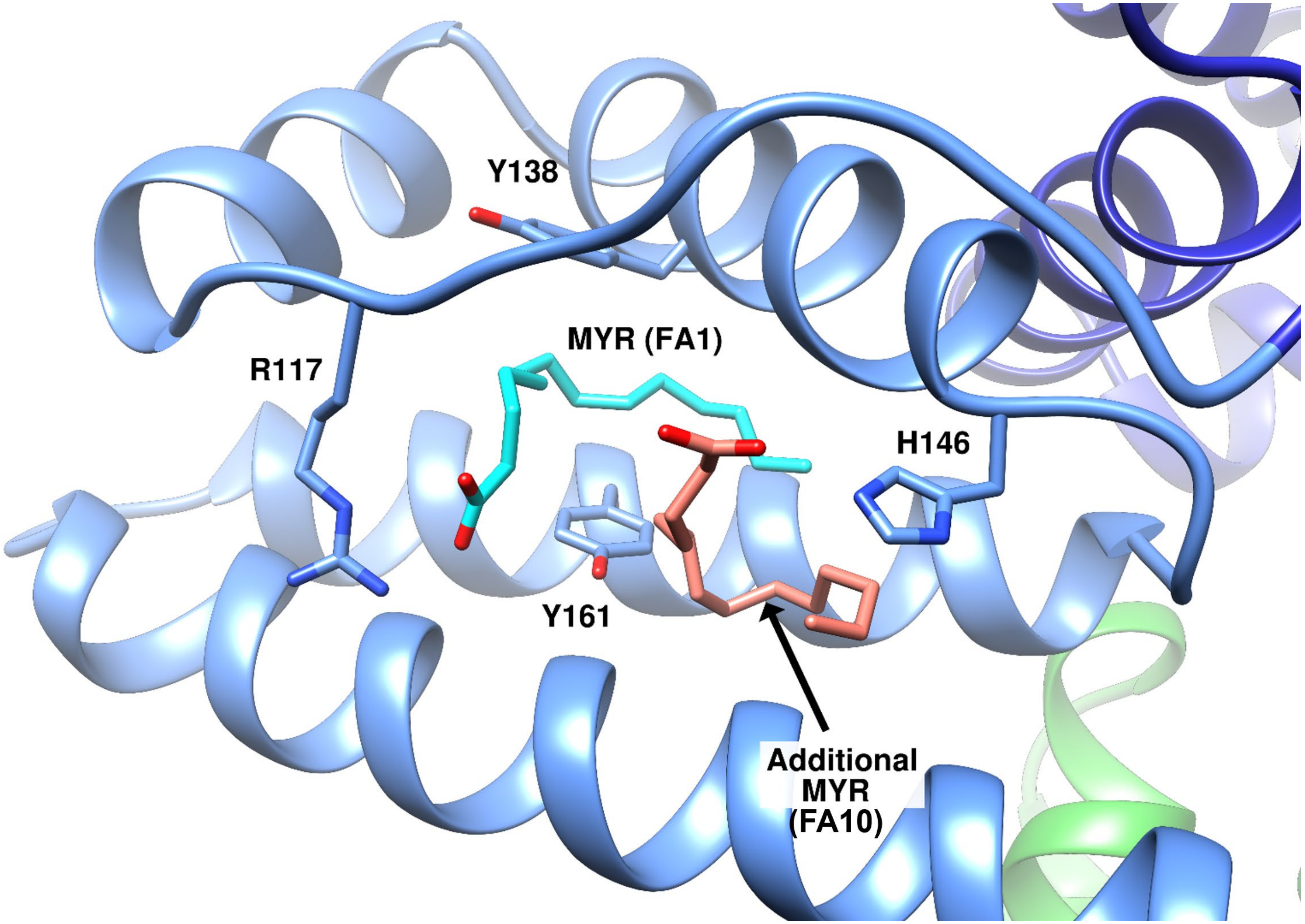

Figure S3. Structure of the HSA_{MYR} Subdomain IB Binding Site and Corresponding Key Amino Acids. The subdomain IB binding cleft adopts an "open" conformation and is occupied by two distinct myristate molecules. One myristate ligand is bound at the typical FA1 position (cyan) deep within the binding site. An additional myristate molecule (FA10) is located at a secondary binding position near the lip of site IB (salmon) in the vicinity of H146.

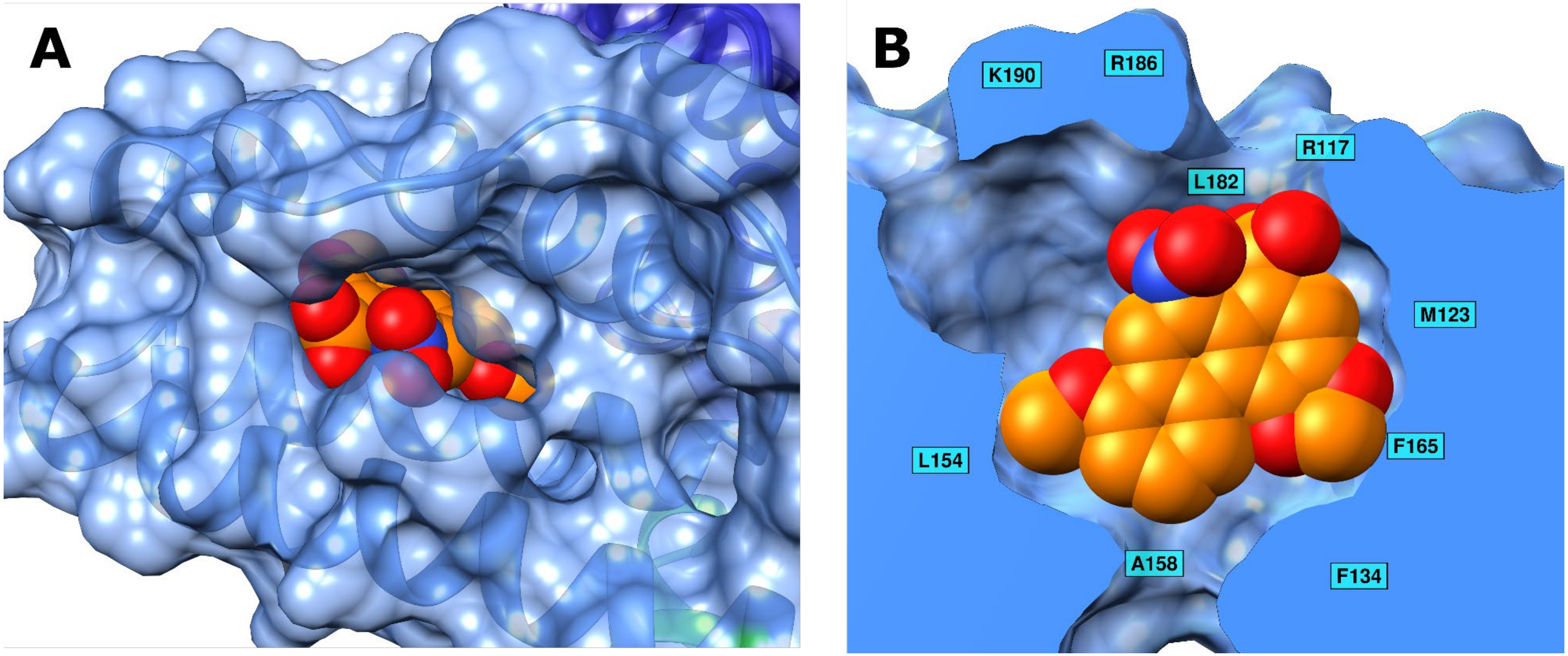

Figure S4. Aristolochic Acid I Bound in the HSA Subdomain IB Site. A: AA-I binds deeply in subdomain IB at the original location of FA1 with only the carboxylate and nitro groups protruding towards solution. **B:** Shape of the subdomain IB binding pocket viewed via cutaway of the HSA molecular surface. AA-I is bound proximate to the inner side where it is surrounded by aliphatic and aromatic residues. The carboxylate and nitro groups point towards a cluster of positively charged resides (e.g., R117, R186) at the "lip" of this pocket.

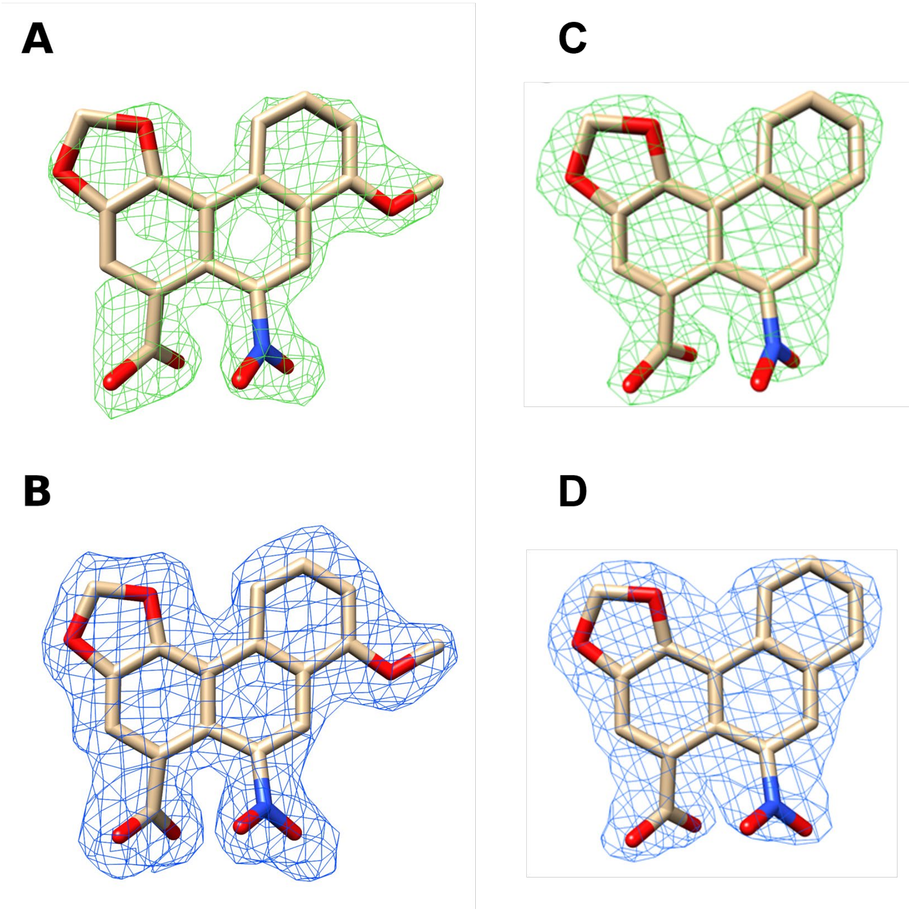

Figure S5. ***Fo-Fc* and *2Fo-Fc* Electron Density Maps of AA-I and AA-II Bound in Subdomains IB and IIA, respectively.** The Fo-Fc (green mesh) (**A**) and *2Fo-Fc* (blue mesh) (**B**) electron density maps of AA-I bound in subdomain IB contoured at 3σ and 1σ, respectively. The *Fo-Fc* (green mesh) (**C**) and 2Fo-Fc (blue mesh) (**D**) electron density maps of AA-II bound in subdomain IIA contoured at 3σ and 1.5σ, respectively.

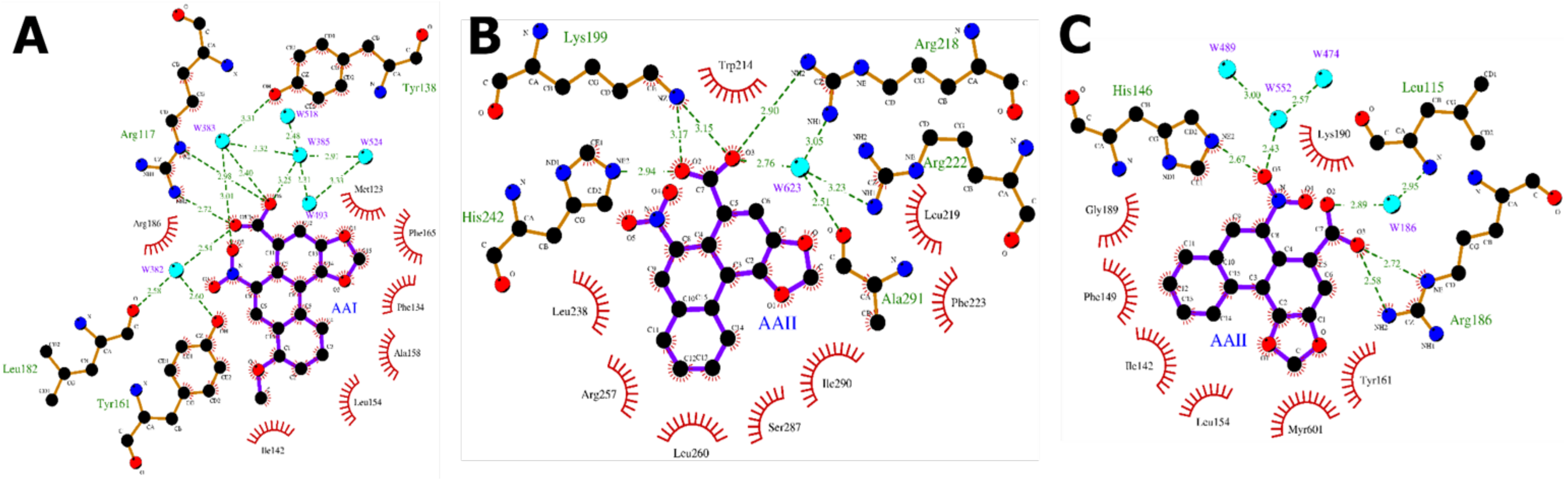

Figure S6. Ligplot Diagrams Depicting Binding Sites in HSA/AA-I (A) and HSA/AA-II (B, C). A: Interactions formed between AA-I and residues of the subdomain IB pocket in the HSA_{MYR}/AA-I complex **B:** Interactions formed between AA-II and residues of subdomain IIA (Drug Site I) in the HSA_{MYR}/AA-II complex **C:** Interactions formed by AA-II with the lip region of site IB in the HSA_{MYR}/AA-II complex. Hydrogen bonds are represented by green dashed lines and hydrophobic contacts by red spokes.

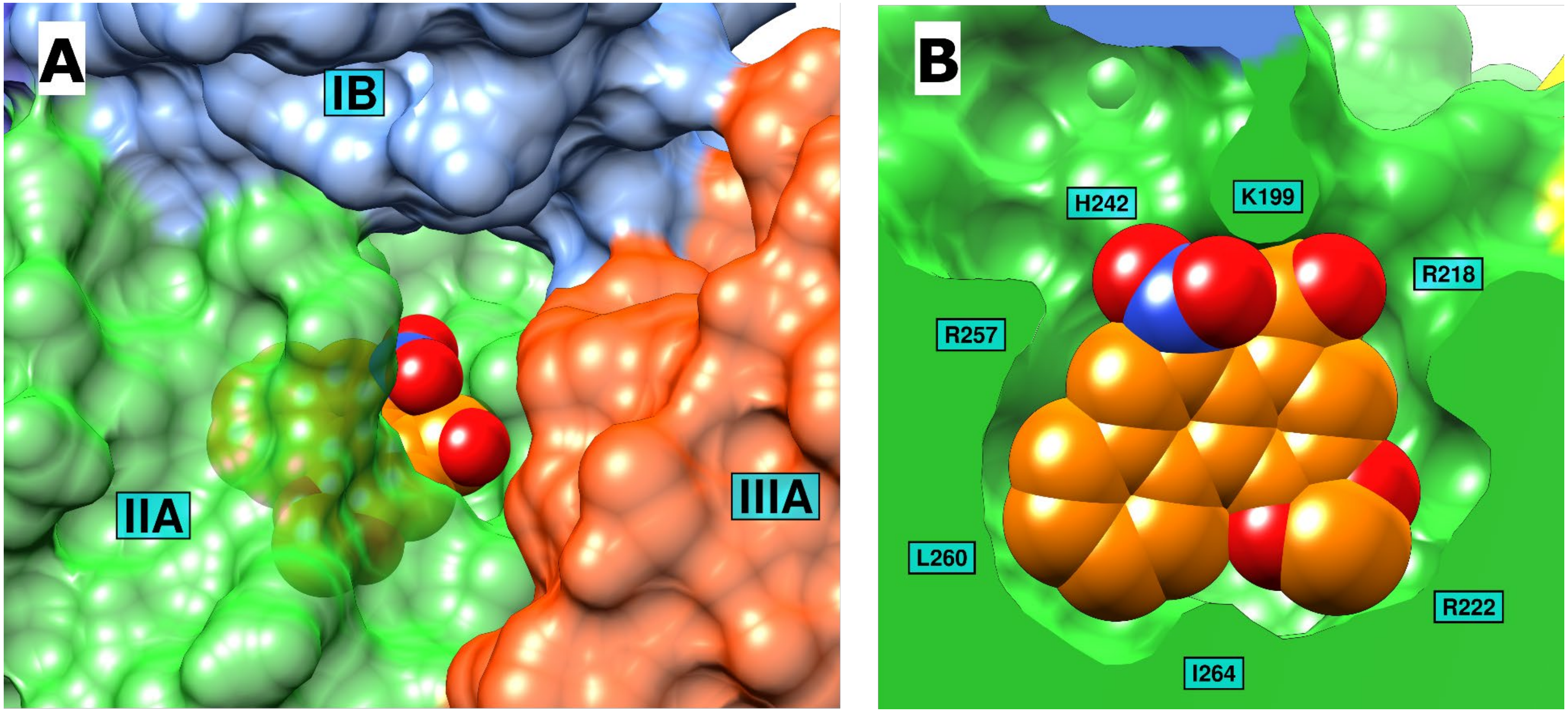

Figure S7. Aristolochic Acid II Bound to HSA Subdomain IIA (Drug Site I). A: AA-II is bound within Drug Site I located in subdomain IIA near the interface between subdomains IB, IIA, and IIIA. Consistent with AA-I, the narrow solvent channel reveals that AA-II is buried within the binding site. **B:** The general shape of Drug Site I and AA-II bound therein viewed via cutaway of the HSA molecular surface. Most of the subdomain IIA pocket is occupied by AA-II with its aromatic section positioned towards the lipophilic region. The carboxylate and nitro groups of AA-II protrude towards two narrow solvent channels and interact with nearby basic residues.

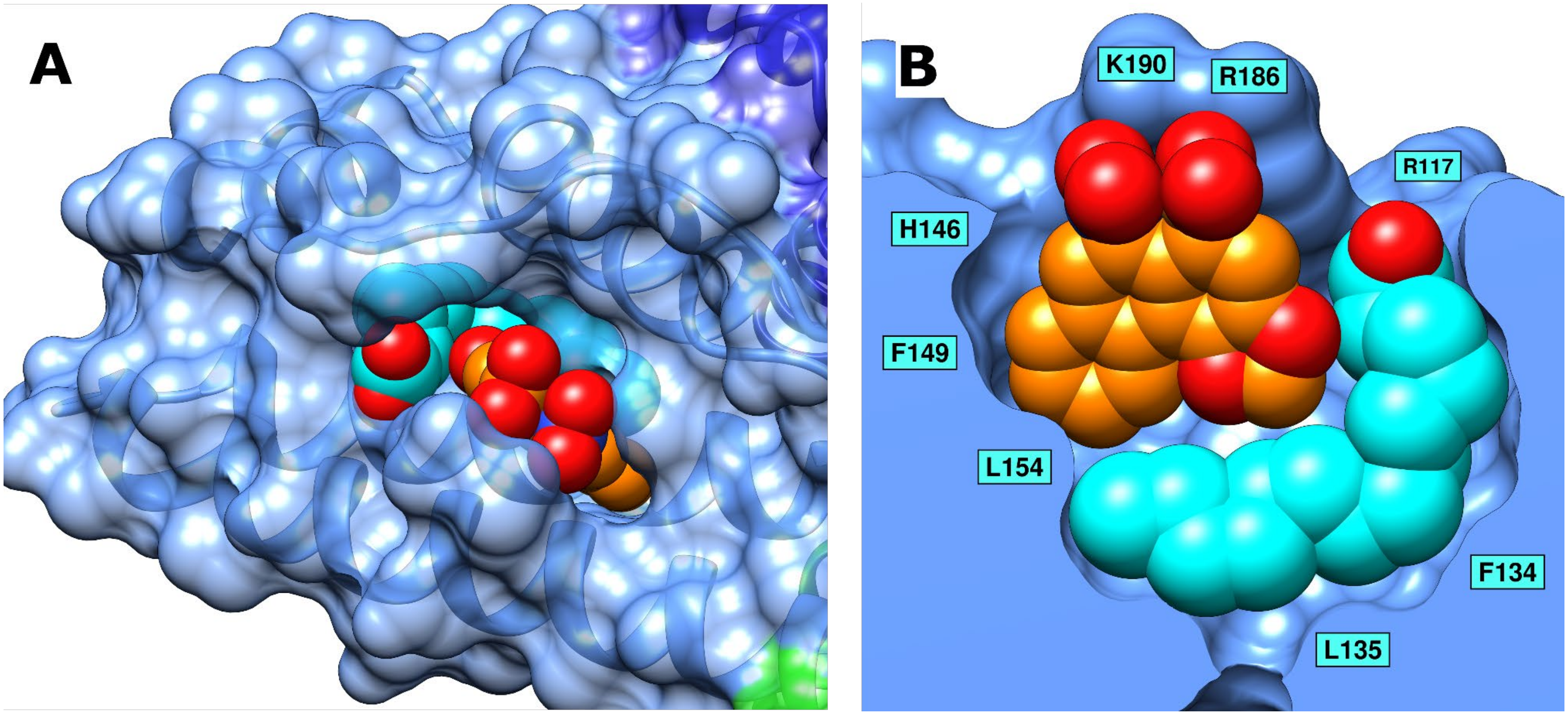

Figure S8. Aristolochic Acid II and FA1 Myristate Bound in HSA Subdomain IB. A: AA-II (orange spheres) and myristate FA1 (cyan spheres) occupy the binding pocket with their carboxylate and nitro groups facing the solvent exposed entrance. **B:** A cutaway view of the HSA molecular surface exposing specific interactions at the subdomain IB binding site. AA-II is bound at the lip of this pocket and a myristate molecule occupies the FA1 position. Both AA-II and FA1 are secured by hydrophobic interactions within the pocket as well as hydrogen bonding and electrostatic interactions at the lip of this binding site.

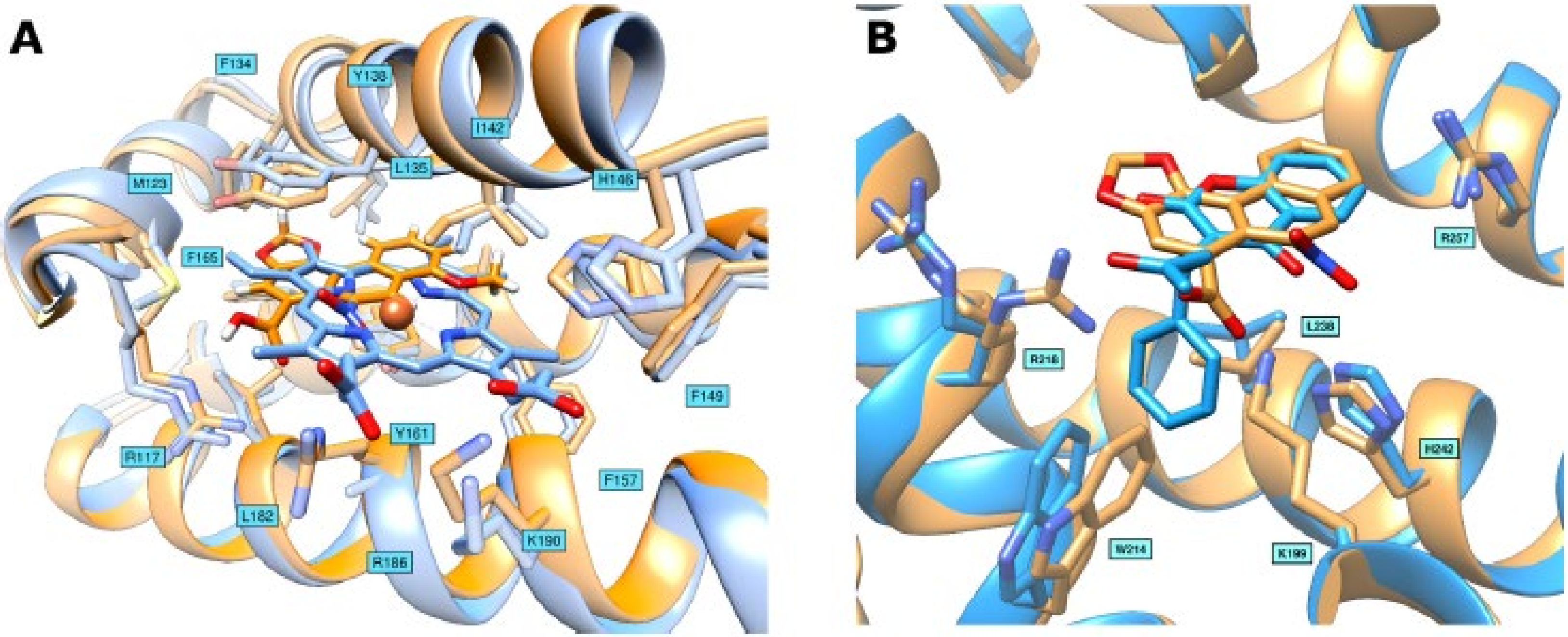

Figure S9. Superimposition of Aristolochic Acids I (A) and II (B) with Ligands that Commonly Bind to HSA Subdomains IB and IIA. A: AA-I (orange) and hemin (light blue) bound in HSA subdomain IB (Drug Site III). Both ligands bind deeply within the subdomain IB pocket as their planar portions engage in hydrophobic and π-stacking interactions while the complex is stabilized by hydrogen bonding and electrostatic interactions with residues at the mouth of this site. **B:** AAII (orange) and warfarin R-(+) (PDB: 1H9Z) (blue) bound in HSA subdomain IIA (Drug Site I). The cyclic moieties of both ligands occupy a similar plane within subdomain IIA that positions their hydrophilic groups in an orientation facilitating hydrogen bonding to basic residues at the entrance of this binding site.

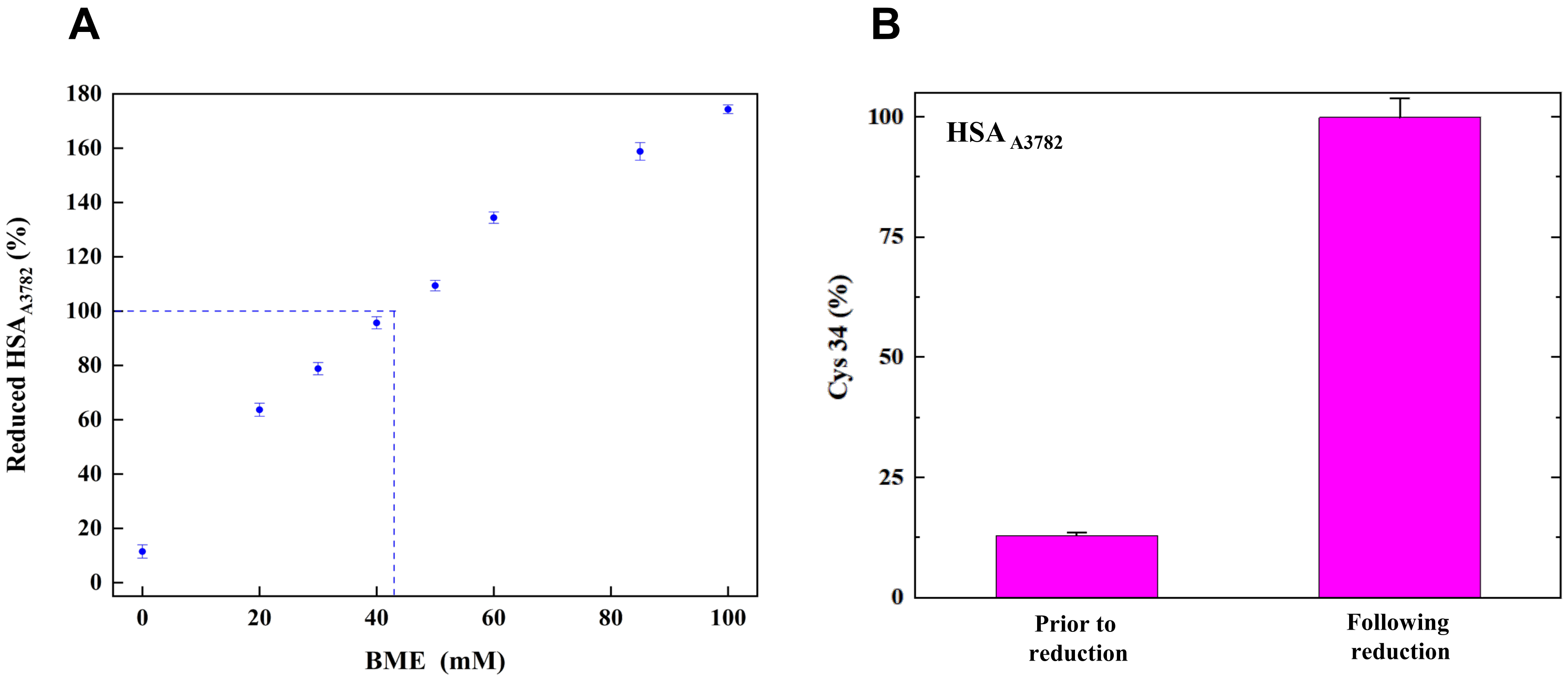

Figure S10. Preparation of BME-reduced CySH34 HSA$_{A3782}$ Stock. A: Small-scale titration of HSA$_{A3782}$ (530 µM) with BME in 20 mM KPO_4 buffer (pH 7.5) monitored via Ellman's assay for quantification of reduced sulfhydryl groups. The resultant linear relation ($Y = 1.4 \cdot X + 38.6$; $r^2 = 0.968$) facilitates selection of the BME concentration (43.98 mM) required for complete Cys34 reduction (~ 92-fold excess) without disrupting internal disulfide bridges. **B:** Evaluation of the HSA CySH34 content prior to and following preparative BME reduction. Under physiological conditions, HSA is comprised of 17 internal disulfide bonds and one free CySH34. Complete reduction of Cys34 without breakage of internal S-S bridges is established when the concentration of total albumin is equivalent to that of CySH34 identified in Ellman's assay.

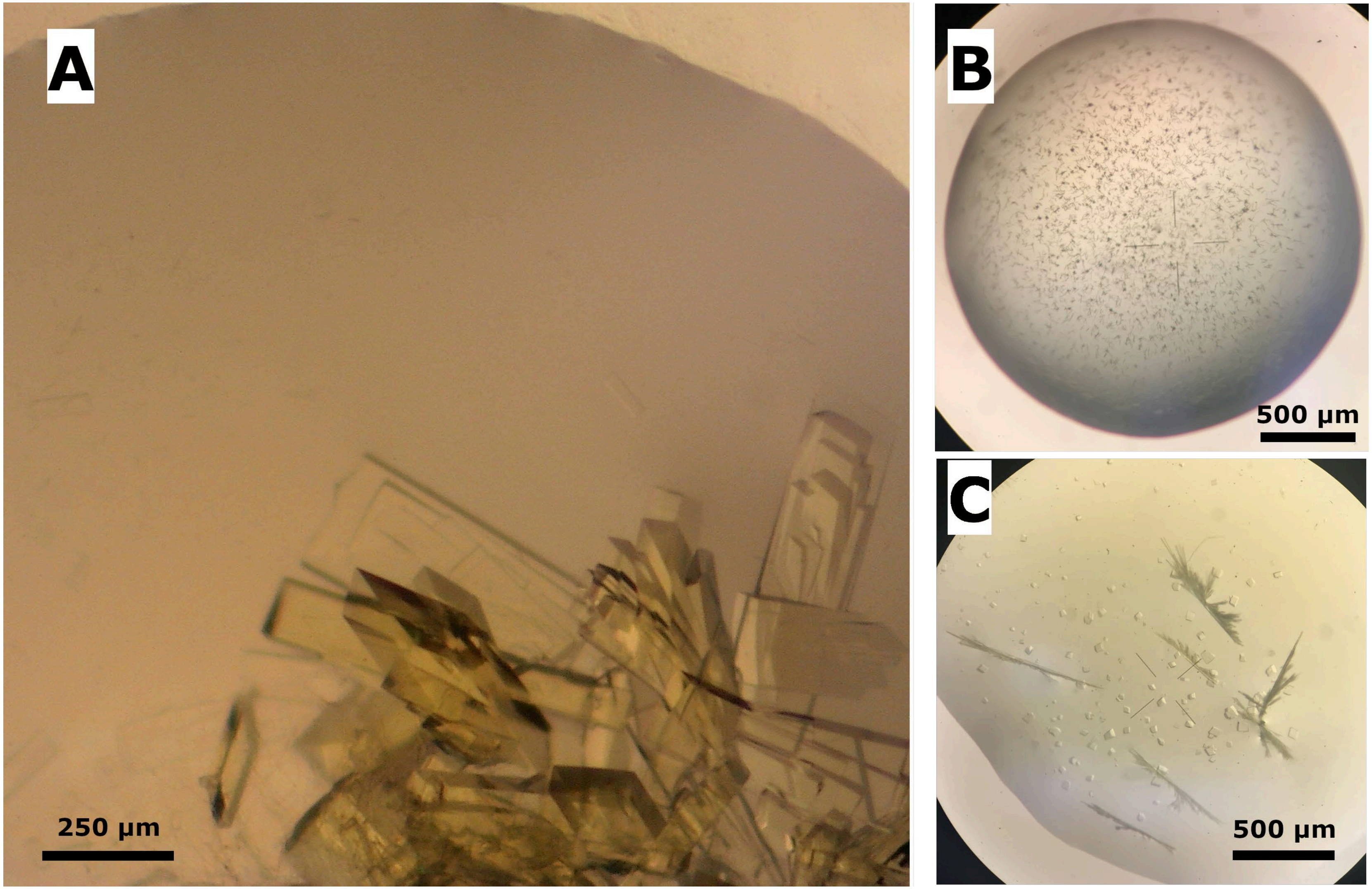

Figure S11. Crystals of HSA, Aristolochic Acids, and HSA/AA Complexes. A: Typical crystals of the HSA_{MYR}/AA-I complex with dimensions on the order of 0.2 x 0.25 x 0.4 mm and a distinct yellow coloration reflecting bound AA-I. **B:** Minute metabolite crystals in an HSA_{MYR}/AL-NOH co-crystallization drop. **C:** Concurrent growth of small rectangular HSA crystals and dark orange metabolite needles under $HSA_{DEFATTED}$/AL-NOH co-crystallization conditions.
